# Transcriptomic profiling of Parkinson's disease brains reveals disease stage specific gene expression changes

**DOI:** 10.1007/s00401-023-02597-7

**Published:** 2023-06-22

**Authors:** Chiara Cappelletti, Sandra Pilar Henriksen, Hanneke Geut, Annemieke J. M. Rozemuller, Wilma D. J. van de Berg, Lasse Pihlstrøm, Mathias Toft

**Affiliations:** 1 Department of Mechanical, Electronics and Chemical Engineering, Faculty of Technology, Art and Design, OsloMet—Oslo Metropolitan University, Oslo, Norway; 2 Department of Research, Innovation and Education, Oslo University Hospital, Oslo, Norway; 3 Department of Neurology, Oslo University Hospital, Oslo, Norway; 4 Amsterdam UMC, Section Clinical Neuroanatomy and Biobanking, Department of Anatomy and Neurosciences, Amsterdam Neuroscience, Vrije Universiteit, Amsterdam, Netherlands; 5 Netherlands Brain Bank, Netherlands Institute of Neurosciences, Amsterdam, Netherlands; 6 Department of Pathology, Amsterdam UMC, Amsterdam Neuroscience, Vrije Universiteit, Amsterdam, Netherlands; 7 Institute of Clinical Medicine, University of Oslo, Oslo, Norway

**Keywords:** Parkinson's disease, Braak Lewy body stage, Human frontal cortex, Neurodegeneration, RNA-sequencing, Gene expression

## Abstract

Parkinson´s disease (PD) is a progressive neurodegenerative disorder characterized by both motor and non-motor symptoms. Aggravation of symptoms is mirrored by accumulation of protein aggregates mainly composed by alpha-synuclein in different brain regions, called Lewy bodies (LB). Previous studies have identified several molecular mechanisms as autophagy and inflammation playing a role in PD pathogenesis. Increased insights into mechanisms involved in early disease stages and driving the progression of the LB pathology are required for the development of disease-modifying strategies. Here, we aimed to elucidate disease stage-specific transcriptomic changes in brain tissue of well-characterized PD and control donors. We collected frontal cortex samples from 84 donors and sequenced both the coding and non-coding RNAs. We categorized our samples into groups based on their degree of LB pathology aiming to recapitulate a central aspect of disease progression. Using an analytical pipeline that corrected for sex, age at death, RNA quality, cell composition and unknown sources of variation, we found major disease stage-specific transcriptomic changes. Gene expression changes were most pronounced in donors at the disease stage when microscopic LB changes first occur in the sampled brain region. Additionally, we identified disease stage-specific enrichment of brain specific pathways and immune mechanisms. On the contrary, we showed that mitochondrial mechanisms are enriched throughout the disease course. Our data-driven approach also suggests a role for several poorly characterized lncRNAs in disease development and progression of PD. Finally, by combining genetic and epigenetic information, we highlighted two genes (*MAP4K4* and *PHYHIP*) as candidate genes for future functional studies. Together our results indicate that transcriptomic dysregulation and associated functional changes are highly disease stage-specific, which has major implications for the study of neurodegenerative disorders.

**Supplementary Information:**

The online version contains supplementary material available at 10.1007/s00401-023-02597-7.

## Introduction

Parkinson's disease (PD) is the second most common neurodegenerative disorder after Alzheimer's disease affecting 2–3% of the population over 65 years of age [69]. The number of PD patients worldwide is anticipated to be more than double by 2040 [28]. There is currently no available disease-modifying treatment to effectively slow the disease process and a better understanding of disease mechanisms is urgently needed to underpin the development of novel therapies.

PD is characterized by neurodegeneration of dopaminergic neurons in the *substantia nigra pars compacta* leading to striatal dopamine depletion, causing motor symptoms such as resting tremor, bradykinesia, and rigidity. The other neuropathological hallmark is the presence of Lewy body (LB) and Lewy neurite (LN) inclusions in remaining neurons and neuronal processes, respectively, mainly composed of aggregates of misfolded alpha-synuclein (α-syn). Historically, a major emphasis has been placed on understanding the neurodegeneration of the *substantia nigra* and the associated motor features of PD. However, it is now widely recognized that PD is a disorder that extends beyond the *substantia nigra*, affecting both the central and peripheral nervous system. Patients also present a range of non-motor symptoms, including prodromal symptoms occurring prior to motor dysfunction [74]. This wider understanding of the clinical features of PD fits well with the recognition of spreading α-syn pathology. In 2003, Braak and colleagues presented a pathological staging system for preclinical and clinical PD based on a specific progression pattern of LB pathology in the brain. Ranging from 1 to 6, the Braak LB stages reflect increasingly widespread presence of LBs in the brain, following a rostral to caudal pattern. The Braak staging system is thought to mirror disease progression, with key clinical symptoms appearing successively as the disease process affects further brain regions [13]. Supporting this notion, approximately 30–60% of the patients develop PD dementia in later stages of the disease, believed to reflect widespread involvement of the neocortex [14, 16].

A small minority of PD patients suffers from a monogenic form of the disease. However, the majority of PD is sporadic, likely caused by a complex interplay between genetic, epigenetic and environmental components, with ageing as the strongest risk factor. Genome wide association studies (GWAS) have discovered a total of 90 genetic loci associated with increased risk for PD [18, 60, 61]. Epigenetic and transcriptomic studies have been performed mainly in blood and *postmortem* brain tissue, the latter arguably being most representative of the core pathology of the disorder. We recently reported four differentially methylated loci associated with LB pathology in an epigenome-wide association study (EWAS) of *postmortem* frontal cortex [68].

A number of transcriptomic studies of *postmortem* PD brain have been published to date, yet their interpretation faces several limitations. Most of the earlier studies used microarray technology, which profiles expression only of the genes and non-coding RNAs included on the array [12, 26]. More recent studies have used RNA-sequencing (RNA-seq), including two that applied the ribosomal RNA depletion method, which allows sequencing of both coding and all non-coding RNAs [35, 45, 63]. This approach has a better and more even coverage of the *postmortem* brain tissue transcriptome as compared to the poly(A) capture method [63]. In general, however, previous PD brain transcriptomic studies have been limited in terms of both sample numbers and selected brain regions. Most studies examined the *substantia nigra*, which shows a 60–80% loss of dopaminergic neurons in PD patients [65], making it very difficult to unravel any pattern of differential expression compared to control brains. Even when studying bulk tissue from parts of the brain where the pathology is less severe, cell composition can be a major confounder of gene expression [63]. Finally, previous studies have predominantly applied a case–control design or investigated the gradually progressive nature of PD using either clinical measures of progression, such as dementia, or by comparing gene expression in brain regions affected at different stages of the disease [21]. However, no study has used neuropathology, the Braak LB staging specifically, as a marker of disease progression.

In this study we analyzed *postmortem* frontal cortex samples from a total of 84 donors including neurologically healthy, incidental LB disease (iLBD), and PD individuals. We categorized our samples into groups based on their pathological Braak LB stage and performed RNA sequencing of both coding and non-coding RNA transcripts. In lack of an established consensus on transcriptomic analyses of brain samples, we developed an analytical pipeline that corrected for sex, age at death, tissue quality, cell composition and unknown sources of variation. We discovered major disease stage-specific transcriptomic changes in the frontal cortex. The changes were most pronounced in donors at Braak LB stage 5, the disease stage when microscopic LB changes are first occurring in the sampled brain region. We identified several poorly characterized lncRNAs as differentially expressed at specific Braak LB stages, suggesting a role for this group of regulatory transcripts in disease development and progression. Additionally, we showed disease stage-specific functional enrichment of brain specific pathways and immune mechanisms, whereas we found that mitochondrial mechanisms are affected throughout the disease course. Our results indicate that transcriptomic dysregulation and associated functional changes are mostly highly disease stage-specific, which has major implications for study design in transcriptomic analyses of neurodegenerative disorders.

## Material and methods

### Subjects and neuropathological assessment

*Postmortem* human brain tissue collected from donors of European ancestry was kindly received from the Netherlands Brain Bank (NBB) (http://www.brainbank.nl/). Standardized brain autopsies and neuropathological examinations were performed by an experienced neuropathologist (A.J.M.R.) or neuroanatomist (W.D.J.v.d.B). LB-related α-syn pathology was assessed according to BrainNet Europe guidelines using tissue from the frontal cortex (Brodmann area 9 or 10) [2] and clinical information was extracted from medical records. PD diagnosis was based on the presence of clinical parkinsonism during life according to UK Parkinson’s Disease Society Brain Bank [36] or Movement Disorders Society [71] criteria, combined with moderate to severe loss of neuromelanin-containing neurons in the *substantia nigra* as well as Lewy pathology in at least the brainstem, with or without limbic and cortical brain regions [24]. A dementia diagnosis was either set by a neurologist or geriatrician during life or assigned retrospectively based on neuropsychological test results showing disturbances in at least two core cognitive domains or Mini-Mental State Examination (MMSE) score < 20 [33].

We obtained tissue from 23 control donors without neurological diseases and 61 individuals with varying degree of α-syn-related pathology. The degree of α-syn-related pathology can be determined by the Braak LB staging system. According to this staging system, LBs first appear in the neurons of the olfactory tract and in the medulla oblongata (stage 1). LBs are then found in the *pontine tegmentum* (stage 2), and emerge in the midbrain, particularly in the *substantia nigra*, in stage 3. LBs are later present in the basal prosencephalon and in the mesocortex (stage 4) and ultimately in increasing portions of the neocortex (stages 5 and 6) [13].

We split the donors with varying degree of α-syn-related pathology into three neuropathological groups based on their Braak LB stage (Supplementary Fig. 1.a). Of note, LBs are not only characteristic of PD, and can also be found during routine *postmortem* examination in the brains of 5–20% of clinically healthy individuals over 60 years old and this finding has been termed iLBD [36, 83]. Individuals with iLBD show moderate neuronal loss in the *substantia nigra* in addition to the presence of LBs corresponding to Braak LB stages 1–2 [27, 48]. Therefore, iLBD presents with nigrostriatal pathological features that are intermediate between neuropathologically normal individuals and PD patients suggesting that iLBD represents presymptomatic PD, instead of a nonspecific, age-related α-syn disease [23, 25]. For this reason, the samples collected from donors for the present study included samples collected from both individuals diagnosed with PD, PDD, and individuals with iLBD (Supplementary Fig. 1.b). Of note, 3 individuals with reported Braak LB stage of 5 were classified as iLBD because no symptoms were recorded. This apparent absence of symptoms in individuals at Braak LB stage 5 may have been caused by a lack of a clinical examination during the last years before death, highlighting the uncertainty of clinical diagnoses. Of note, dementia with Lewy bodies (DLB) was used as an exclusion criterion (Supplementary text).

### Sample preparation

Frozen tissue blocks from the superior frontal *gyrus* at the level of *anterior cornua* of lateral ventricles corresponding to Brodmann area 8 or 9 were collected at autopsy and stored at − 80 °C until further processing in the NBB. To obtain grey matter tissue for the present study, tissue blocks were mounted in a cryostat and thin tissue sections of 50–100 mg spanning all cortical layers were carefully sliced from the block by a skilled technician and collected in the tubes used for nucleic acid extraction. DNA and total RNA were simultaneously isolated from brain frontal cortex tissue with the AllPrep® DNA/RNA/miRNA Universal kit (#80,224 Qiagen, Germany) according to the manufacturer’s instructions to achieve maximum yields of both DNA and RNA. The RNA concentration and purity were analyzed using the NanoDrop-1000 Spectrophotometer (Thermo Fisher Scientific). For each sample we obtained 6–18 μg RNA. The RNA integrity number (RIN) was assessed using the RNA Nano 6000 Assay Kit of Bioanalyzer 2100 system (Agilent Technologies, CA, USA). RNA samples had a mean RIN = 7.8, SD = 0.7 (range 5.4–9.0). RNA samples were stored at − 80 °C.

### RNA-sequencing and quality control

2 μg of total RNA was used for downstream RNA-seq applications performed by Novogene (Novogene Biotechnology Inc, Beijing, China) on the NovaSeq 6000 platform. The NEBNext Ultra RNA Library Prep Kit for Illumina was used for library preparation. Both cytoplasmic and mitochondrial rRNAs were removed using the Ribo-Zero Gold kit from Illumina. The library was paired-end sequenced (2 × 150 bp) to a depth of 50 million reads (25 million reads paired-end fragments). FASTQ files were assessed using fastQC version 0.11.9 [4] and MultiQC version 1.8 [34] with default settings prior to alignment and quantification.

### RNA expression quantification and filtering

Salmon version 1.3.0 [67] was used to quantify the abundance at the transcript level with the options: (1) sequence-specific bias (–seqBias), (2) fragment-level GC bias (–gcBias), (3) –validateMapping, and (4) appropriate library type (-l ISR). The transcript abundance was quantified against the Genecode v35 transcriptome (corresponding to Ensembl release 101 transcriptome) (Supplementary Fig. 2.a). Transcript-level quantification was collapsed onto gene-level quantification using the tximport R package version 1.20.0 [76].

### Cell deconvolution

A limitation of bulk-tissue RNA-seq is that only the average gene expression levels across many molecularly diverse cell types are captured [31]. Consequently, a difference in gene expression levels between experimental groups could be caused by a variation in cellular composition between the samples or a change in gene expression in a specific cell population, or a combination of the two. Moreover, RNAs from different cell types may be differentially susceptible to degradation. Therefore, when studying neurodegenerative diseases such as PD, where neuronal loss may lead to systematic cell populations differences between experimental groups, it is particularly important to deconvolve the cell-type composition from a change in gene expression [49].

We first estimated cell-type proportions using Scaden version 1.1.2 [76]. Scaden is a deep-learning based deconvolution algorithm that is trained on artificial bulk-tissue RNA-seq samples simulated from tissue specific single-cell RNA-seq (scRNA-seq) data. It then uses the generated model to predict cell-type proportions from real bulk-tissue RNA-seq samples. In this study, we used the provided mouse brain training data. This training data was obtained by simulating artificial bulk RNA-seq samples from five scRNA-seq datasets obtained from different mouse brain regions [17, 20, 73, 80, 89], and has been validated for the use in the human brain frontal cortex by Menden and colleagues [58]. Our RNA-seq samples were used in the form of counts normalized to library size using the median ratio method [3] in DESeq2 (version 1.34.0) [54] including only genes with count > 0 in all samples and excluding mitochondrial genes (21,615 genes). To ensure the training data and the RNA-seq samples for prediction shared the same genes and feature scale, first the human gene symbols were converted to mouse gene symbols (14.603 genes) and then both the training and the RNA-seq datasets were pre-processed with Scaden process. The two datasets shared a total of 11.754 genes. The Scaden model was then trained (Scaden train) using default setting for 5000 steps as recommended by the developers to prevent overfitting. Lastly, predictions for cell-type proportions were made with Scaden predict.

To confirm the results obtained with Scaden we estimated the cell composition of our samples also using marker gene profiles (MGPs) [55]. We employed a marker gene list, containing marker genes specific for 17 cell types, obtained from scRNA-seq data of the human prefrontal cortex and *anterior cingulate* cortex (Supplementary text) [84].

The pairwise Pearson's correlation between cell proportions and potential sources of biological variation in our data (neuropathological group, sex, age at death, *postmortem *delay (PMD) and RIN was investigated using the cor_mat() R function. Linear models adjusting for known experimental covariates (sex, age at death, PMD and RIN) were used to examine the differences in cell composition between the neuropathological groups.

### Quality surrogate variable analysis

We used the quality surrogate variable analysis (qSVA) framework to estimate and remove RNA quality confounding in differential expression analysis. First reads were mapped to the GRCh38 human reference genome with HISAT2 version 2.2.1 [46] using –rna-strandness RF option (Supplementary Fig. 2.b). Samtools version 1.12 [53] was used to generate BAM files. We used the 515 degradation-susceptible regions identified by Jeffe et al. [42] in superior prefrontal cortex samples obtained from data generated with the RiboZero protocol and our BAM files to generate the degradation matrix (defined as the library-size normalized coverage across each of these regions in our dataset) with the python script region_matrix.py provided by the authors. The read.degradation.matrix() function implemented in the sva package version 3.43.0 [52] was used to normalize the degradation matrix to a common library size of 80 M. The quality surrogate variables (qSVs) were estimated using the qsva() function implemented in the sva package. The pairwise Pearson's correlation between five qSVs, known sources of RNA quality variation (PMD and RIN) and cell-type proportions was investigated using the cor_mat() R function. Five qSVs were included in further analysis as covariates.

### Covariate selection

Principal component analysis (PCA) was performed on gene-level expression counts filtered to include only genes with count > 0 in all samples and excluding mitochondrial genes (21,615 genes), and transformed with vst() function from DESeq2 [54], which applies a variance stabilizing transformation, to explore the effect of accounting for sex, age at death and the five qSVs on variation in RNA-seq data. Samples were plotted by their first two principal components derived from uncorrected gene expression (Supplementary Fig. 3.a) and gene expression adjusted for sex, age at death and five qSVs (Supplementary Fig. 3.b) to determine how well the neuropathological groups separated. Count correction was performed using the removeBatchEffect() function from the R package limma (3.50.0) [75].

### Differential gene expression analysis

Differential gene expression between the neuropathological groups was assessed using the DESeq2 R package (version 1.34.0) [54]. Mitochondrial genes were excluded and only genes with count > 0 in all samples (21,615 genes) were used in the analysis. Gene expression analysis was performed controlling for covariates (sex, age at death and five qSVs). The PD samples in the different Braak LB groups are from disease stages ordered in a progressive manner. We first wanted to identify gene groups whose expression patterns change along the progressive course of increasing LB pathology using the likelihood ratio test (LRT). Subsequently, the Wald test was used for the pairwise comparison of Braak LB stages 1–4, 5 and 6 to Braak LB stage 0. All analyses were followed by false discovery rate (FDR) calculation by the Benjamini and Hochberg procedure.

### Expression pattern analysis

To investigate expression trajectories across disease stages, gene expression levels of the differentially expressed genes in the main analysis were scaled to z-scores and clustered using the divisive hierarchical clustering implementation of the degPatterns function from the R package DEGreport (1.30.0) [66]. The Kendall rank correlation coefficient was used as distance metric.

### Functional enrichment analysis

Genes were scored by transforming the *p*-values obtained from the pairwise comparisons (Braak LB stage 0 vs 1–4, Braak LB stage 0 vs 5, and Braak LB stage 0 vs 6) to account for direction of change in expression as previously described [63]. By choosing to take into account only the direction of the fold changes, this method prevents the effect of single outliers. Gene sets were then tested for enrichment using either the log(Score_UP_) or the log(Score_DOWN_) scores with the gene score resampling method part of the ermineR [56], an R wrapper package for ermineJ [50], with the complete Gene Ontology database annotation [6], to obtain a list of up- and down-regulated enriched pathways. To compare the biological pathways enriched at Braak LB stage 1–4, 5 and 6, we quantified the difference in the level of significance in the up- and down-regulated results for each significant pathway as Δ_1_ = log(p_1-4_)—log(p_5_) and as Δ_2_ = log(p_5_)—log(p_6_) were p_1-4_, p_5_ and p_6_ are the corrected enrichment *p*-values for the different comparisons (1–4 = Braak LB stage 0 vs 1–4, 5 = Braak LB stage 0 vs 5 and 6 = Braak LB stage 0 vs 6). Only pathways that were significant in either one of the comparisons were included in the analysis (p_1-4_ < 0.05, p_5_ < 0.05 or p_6_ < 0.05). (Results presented in supplementary text). Similar pathways were summarized by combining the Gene Ontology terms that share many genes as previously described [63].

### Expression quantitative trait locus analyses

The list of 90 risk SNPs for PD identified in the largest GWAS, including information on the nearest gene and genes nominated based on QTLs, were extracted from Nalls et al. [60]. 87 of the 97 genes highlighted in the GWAS were expressed in our samples. 83 samples had been previously genotyped using the NeuroChip array (Illumina, San Diego, CA USA) [10, 68]. The association between the adjusted expression of identified risk genes and the genotype of their nearby SNP were tested using a linear regression model (lm() function in R).

### Investigation of genes in differentially methylated loci

Four genes (*TMCC2*, *SFMBT2*, *AKAP6* and *PHYHIP*) located near the differentially methylated replicating loci associated with Braak LB stage reported by Pihlstrøm et al*.* [68] were manually searched among the 266 genes differentially expressed between Braak LB stage 0 and Braak LB stage 5.

## Results

### Gene expression in the superior frontal gyrus of individuals at different pathological disease stages

**Fig. 1:**
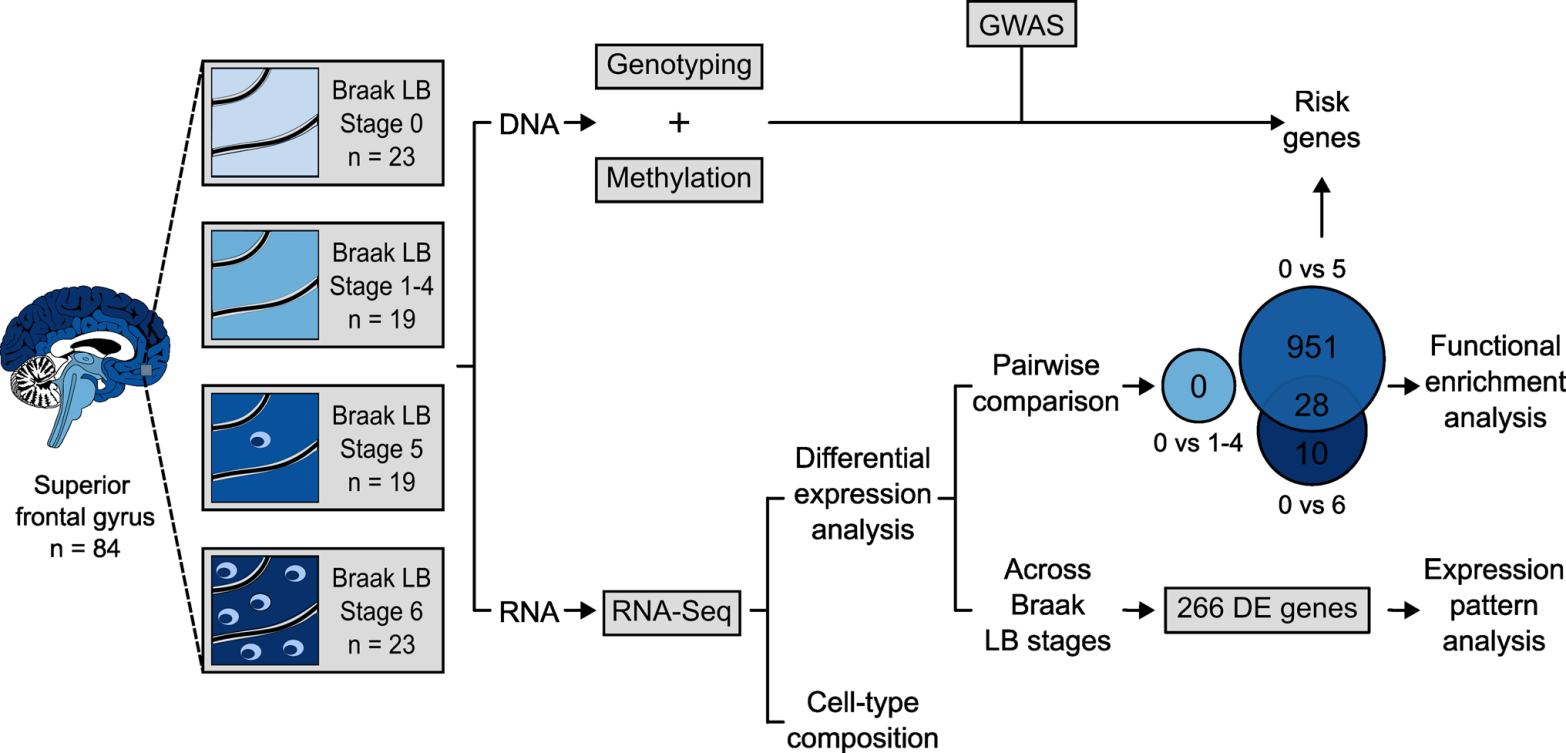
Study overview. In this study, the frontal cortex was sampled in a cohort of 84 individuals divided into four neuropathological groups based on their Braak LB stage: non-neurological controls, Braak LB stage 1–4, Braak LB stage 5, and Braak LB stage 6. DNA and RNA were simultaneously isolated from each sample. DNA was used for genotyping and methylation analysis [68]. RNA was sequenced and data used to estimate cell-type composition and to perform differential expression analysis. Differential expression analysis comparing the transcriptome across all neuropathological groups identified 266 genes differentially expressed (FDR < 0.05) that were used for expression pattern analysis. Differential expression analysis comparing Braak LB stage 1–4, 5 and 6 to Braak LB stage 0 in a pairwise manner found 0 differentially expressed genes at Braak LB stage 1–4 (FDR < 0.05), 979 differentially expressed genes at Braak LB stage 5 (FDR < 0.05), and 38 differentially expressed genes at Braak LB stage 6 (FDR < 0.05). Results from this analysis were used for functional enrichment analysis. Genes DE at Braak LB stage 5 were combined with the genotyping data for the 90 risk PD loci identified by GWAS to perform eQTL analysis

In this study, we examined the differences at the transcriptome level in the superior frontal cortex of 84 individuals, including 23 non-neurological controls and 61 individuals affected by α-syn-related pathology at different Braak LB stages. The latter were split into three neuropathological groups based on their Braak LB stage: one group consisting of individuals without LB pathology in the analyzed tissue, corresponding to Braak LB stages 1–4, (*n* = 19), one group consisting of individuals with moderate LB pathology in the frontal cortex (Braak LB stage 5, *n* = 19) and one group consisting of individuals with a higher load of frontal cortex LBs (Braak LB stage 6, *n* = 23) (Supplementary Fig. 1.a, Supplementary Table 1). Of note, RNA was extracted from the superior frontal cortex where LBs become present at Braak LB stage 5 [13] (Fig. 1).

**Fig. 2:**
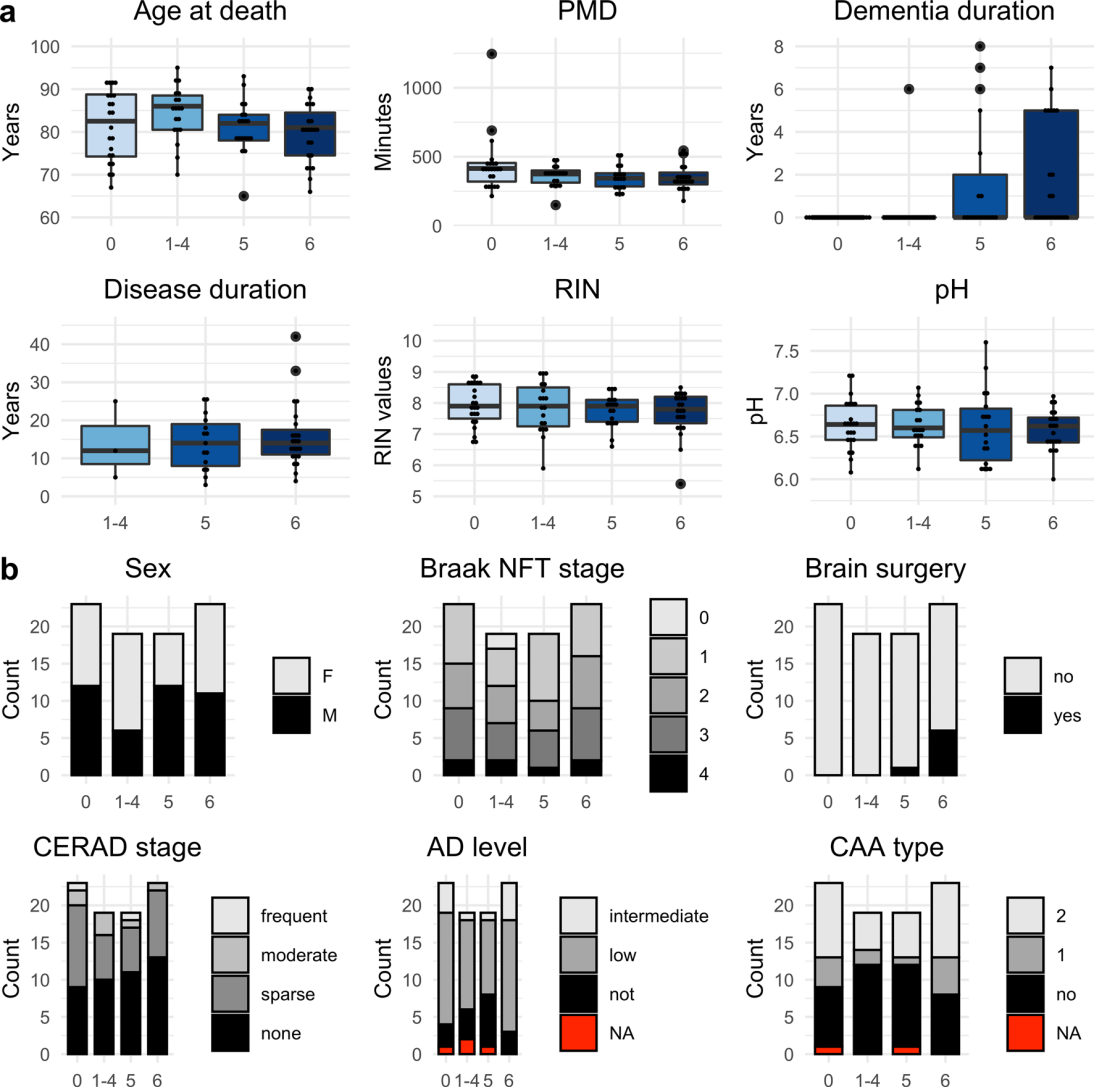
Individuals’ demographics, sample information and pathological measures. **a** Continuous and **b** categorical individuals’ demographics, sample information and pathological measures are shown for each neuropathological group. Significant differences between neuropathological groups were tested using either the Kruskal–Wallis rank sum test (for continuous variables) or the Chi-squared test (for categorical variables). *PMD* *postmortem* delay, *RIN* RNA integrity number, *NFT* neurofibrillary tangle, *CERAD* consortium to establish a registry for Alzheimer's disease, *AD* Alzheimer's disease, *CAA* cerebral amyloid angiopathy

The selected individuals were matched for demographic factors (sex and age at death) and levels of Alzheimer’s disease (AD) related pathology (Fig. 2 and Supplementary Table 1). A significant difference between the groups was observed for the proportions of individuals who had undergone deep brain stimulation surgery (Braak LB 0 = 0/23, Braak LB 1–4 = 0/19, Braak LB 5 = 1/19, Braak LB 6 = 6/23, *p*-value = 0.0036, Chi-squared test). This difference is explained by the fact that deep brain stimulation may be a treatment option in selected patients at more advanced stages of the disease. The duration of dementia also differed between groups, with groups 5 and 6 having had dementia for a longer time (*p*-value = 0.0002, Kruskal–Wallis rank sum test). This difference can be attributed to the fact that dementia appears in advanced stages of the disease. Of note, no differences were found between the groups for the Braak neurofibrillary tangle stage, the composite “ABC-score” reflecting total AD-related pathology [41], the CERAD stage or the CAA type, indicating that concomitant AD pathology was not an important confounding factor in our analysis. Moreover, for all the samples the *postmortem* delay was very short (mean = 377 min, SD = 135 min) ensuring high sample quality.

We performed RNA-seq following ribosomal RNA depletion and we detected a total of 21,615 genes expressed in all samples, including long non-coding RNAs (lncRNAs).

### Cell composition and RNA quality

**Fig. 3:**
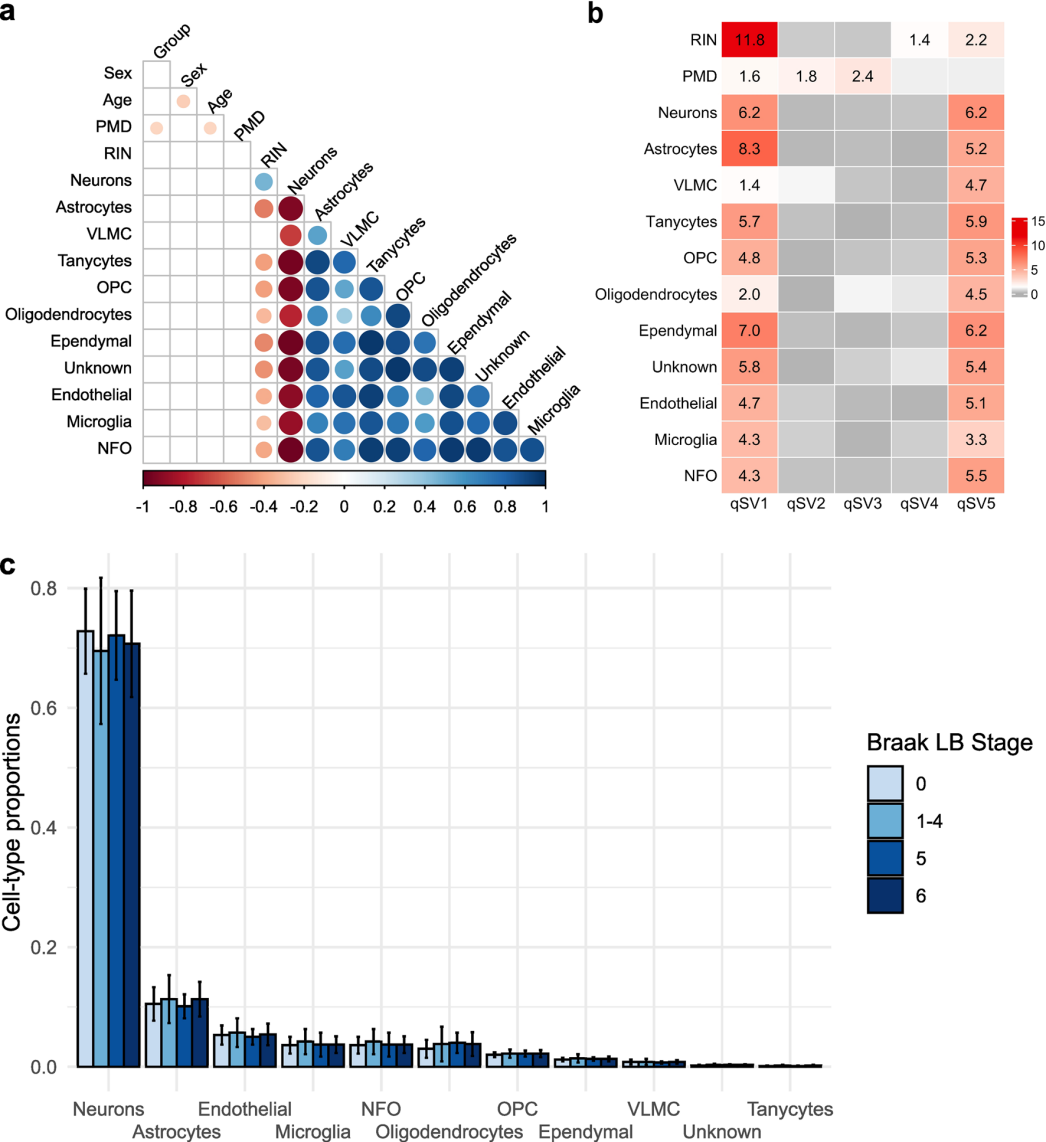
Cell composition and RNA quality. **a** Pearson's correlation between cell-type proportions and potential sources of biological variation. Circles’ sizes are proportional to the Pearson correlation coefficient, with color indicating positive (blue) or negative (red) coefficients. Non-significant pairwise correlations (*p* > 0.05) are not shown. **b** Heatmap showing the association between known variables associated with RNA quality with the five qSVs. Only significant *p*-values (− log10 *p*-value > 1.3) are shown. **c** Cell-type proportions for each neuropathological group. *RIN* RNA integrity number, *PMD* *postmortem* delay, *VLMC* vascular and leptomeningeal cell, *OPC* oligodendrocyte progenitor cell, *NFO* newly formed oligodendrocyte

In our study we estimated cell-type proportions (Supplementary Table 2) using Scaden [58]. We inspected the Pearson's correlation between cell-type proportions and potential sources of biological variation in our data (neuropathological group, sex, age at death, PMD and RIN). The proportion of neurons was significantly anticorrelated with the other cell-type proportions (*p* < 0.05) (Fig. 3a). Moreover, in line with previous studies [8, 42, 63], cell-type proportions were correlated with RIN values (positive correlation with proportions of neurons, negative correlation with other cell-type proportions) (Fig. 3a). No association was found between the cell-type proportions and the other variables (neuropathological group, sex, age and PMD) (Fig. 3a). We then looked for differences in cell-type proportions between the neuropathological groups adjusting for the known experimental covariates (sex, age at death, PMD, RIN). No significant differences (*p*-value > 0.2) were found among the four neuropathological groups (0, 1–4, 5 and 6) for any cell type (Fig. 3b). To confirm this result, we estimated cell composition in our samples also using marker gene profiles [55, 84] and found no significant difference (*p*-value > 0.3) for any cell type between the neuropathological groups (0, 1–4, 5 and 6) (Supplementary Fig. 4).

Since the RNA quality is correlated with cell-type proportions and it has also been shown to influence gene expression levels measurements [1], we used the quality surrogate variable analysis (qSVA) framework to estimate RNA quality confounding. To investigate the variance captured by the five quality surrogate variables (qSVs) obtained with this method we inspected the Pearson's correlation between them and the known variables related to RNA quality (RIN and PMD). Moreover, since we had shown that cell proportions are significantly correlated with RNA quality measured by the RIN values, we examined also the Pearson's correlation between the five qSVs and the cell-type proportions. We found that qSV1 was significantly correlated with all the variables analyzed. Moreover, qSV5 was significantly correlated with all the variables except PMD, which was the only variable associate with qSV2 and qSV3. Lastly, qSV4 was significantly correlated with the RIN values (Fig. 3.c). Therefore, we included five quality surrogate variables (qSVs) as covariates instead of using the samples' RIN values and PMD.

### Differential gene expression across neuropathological stages

To identify groups of genes whose expression levels change along with the progression of LB neuropathology we performed differential gene expression analysis of a total of 21,615 transcripts across all four neuropathological groups. Sex, age at death and five qSVs were used as experimental covariates in LRT tests. The null hypothesis is that gene expression is stable across groups, and the test is indifferent to group order, potentially detecting non-stable expression patterns with any possible combination of up- and downregulation. The differential gene expression analysis found evidence for a total of 266 genes differentially expressed across the four neuropathological groups (LRT test, Benjamini–Hochberg FDR < 0.05) of which 34 corresponded to lncRNAs (Supplementary Table 3).

**Fig. 4:**
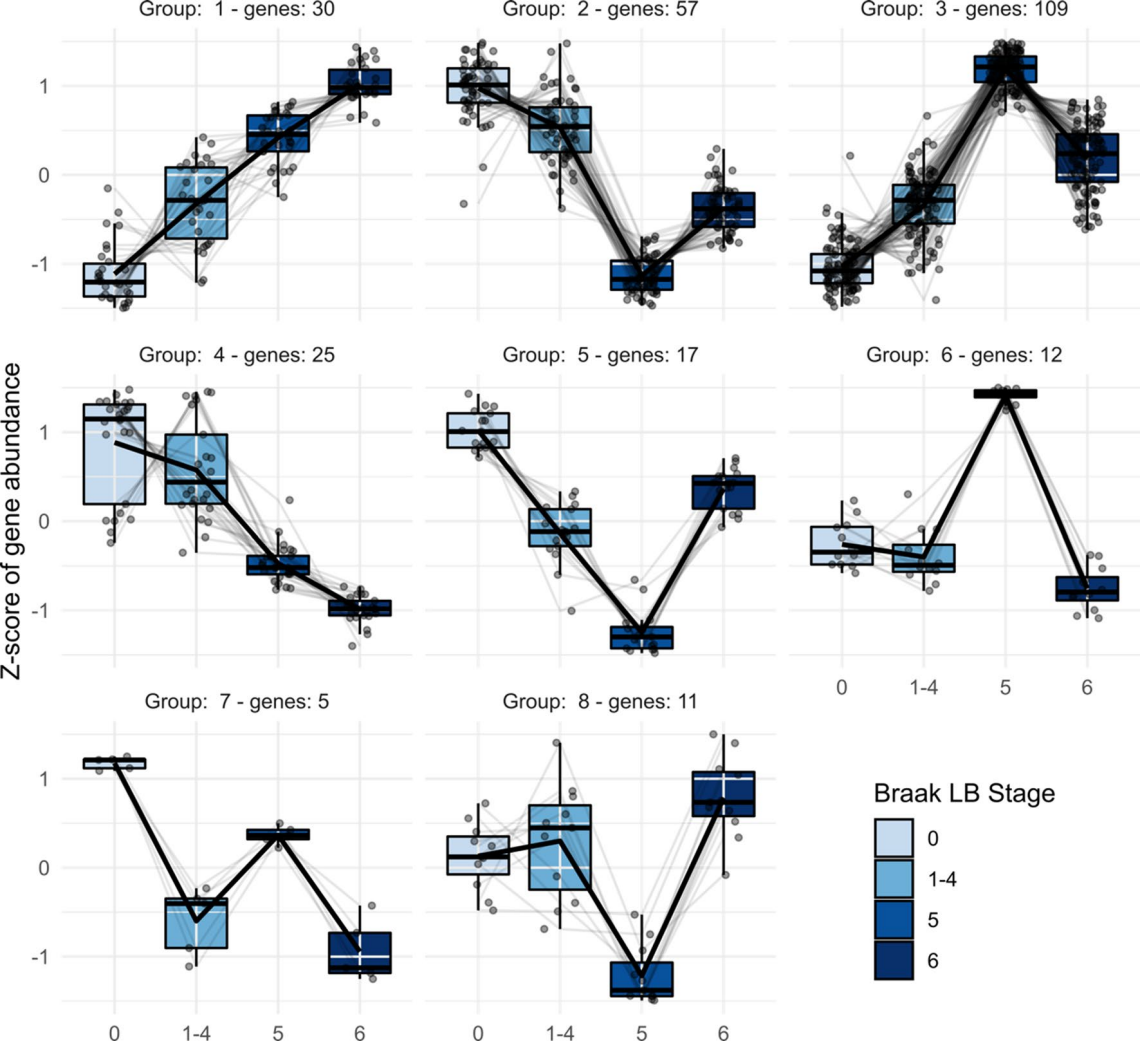
Clusters of differentially expressed genes across Braak LB stages. Divisive hierarchical clustering was performed for 266 differentially expressed genes (FDR < 0.05) according to log2 normalized read counts. Each cluster's number is provided together with the number of genes included in that cluster. Genes are plotted on the y-axis according to the scaled expression value (z-score). Lines connect the mean expression level at consecutive Braak stages to display an expression path typical of that cluster

To further investigate these 266 differentially expressed genes, their regularized log2 transformed counts were clustered using the divisive hierarchical clustering implemented in the DEGreport R package [66]. The differentially expressed genes were divided in 8 clusters (Supplementary Table 3). The expression pattern across the neuropathological groups typical for each of these clusters, together with the scaled expression level of each individual gene, is shown in Fig. 4.

Clusters 1 (30 genes) and 4 (25 genes) showed a pattern of linear increase and decrease in expression from Braak LB stage 0 to stage 6, respectively. The top hit gene of the up-regulated cluster (1) was *SNX7* (Supplementary Fig. 5a), whereas the top hit of the down-regulated cluster (4) was *NUCB1* (Supplementary Fig. 5b). All the other clusters exhibited a pattern of expression in which samples at Braak LB stage 1–4 and 6 had relatively similar expression levels, whereas samples at Braak LB stage 5 showed major changes in gene expression levels when compared to the other groups. Among these clusters showing major transcriptomic changes, clusters 2 and 3 comprised most of the genes (57 and 109, respectively). The top hit gene of cluster 2 is *PDXK* (Supplementary Fig. 5c), whereas the three most significant genes included in cluster 3 are novel transcripts. Another way to visualize how gene expression distinguished Braak LB stage 5 from the other neuropathological groups is presented in the heatmap included in the supplementary material (Supplementary Fig. 6).

### Pairwise differential gene expression between Braak LB stage 0 controls and Braak LB pathology groups

To further investigate the major changes in gene expression of samples at Braak LB stage 5 we compared the transcriptome of this group to the transcriptome of the control group (Braak LB stage 0). Differential gene expression analysis using Wald test of a total of 21,615 genes was performed using sex, age at death and five qSVs as covariates. We found a total of 979 genes differentially expressed (Benjamini–Hochberg FDR < 0.05) between samples at Braak LB stage 0 and at Braak LB stage 5 of which 575 were up-regulated and 404 were down-regulated (Supplementary Table 4). Of the 979 genes differentially expressed at Braak LB stage 5, 233 genes were among the 266 genes differentially expressed when comparing gene expression across all the Braak LB stage groups in the main analysis. Of note, no differentially expressed genes at FDR < 0.05 were identified when comparing groups 0 and 1–4 and 38 genes were significantly differentially expressed between Braak LB stage 0 and 6 (Supplementary Table 5). 28 of these 38 genes differentially expressed between Braak LB stage 0 and 6 were also among the differentially expressed genes between Braak LB stage 0 and 5. The top hit gene at both Braak LB stage 5 and 6 was the *SNX7* gene. These findings further highlight that the most pronounced changes in gene expression of frontal cortex can be observed at Braak LB stage 5.

### Functional enrichment

**Fig. 5:**
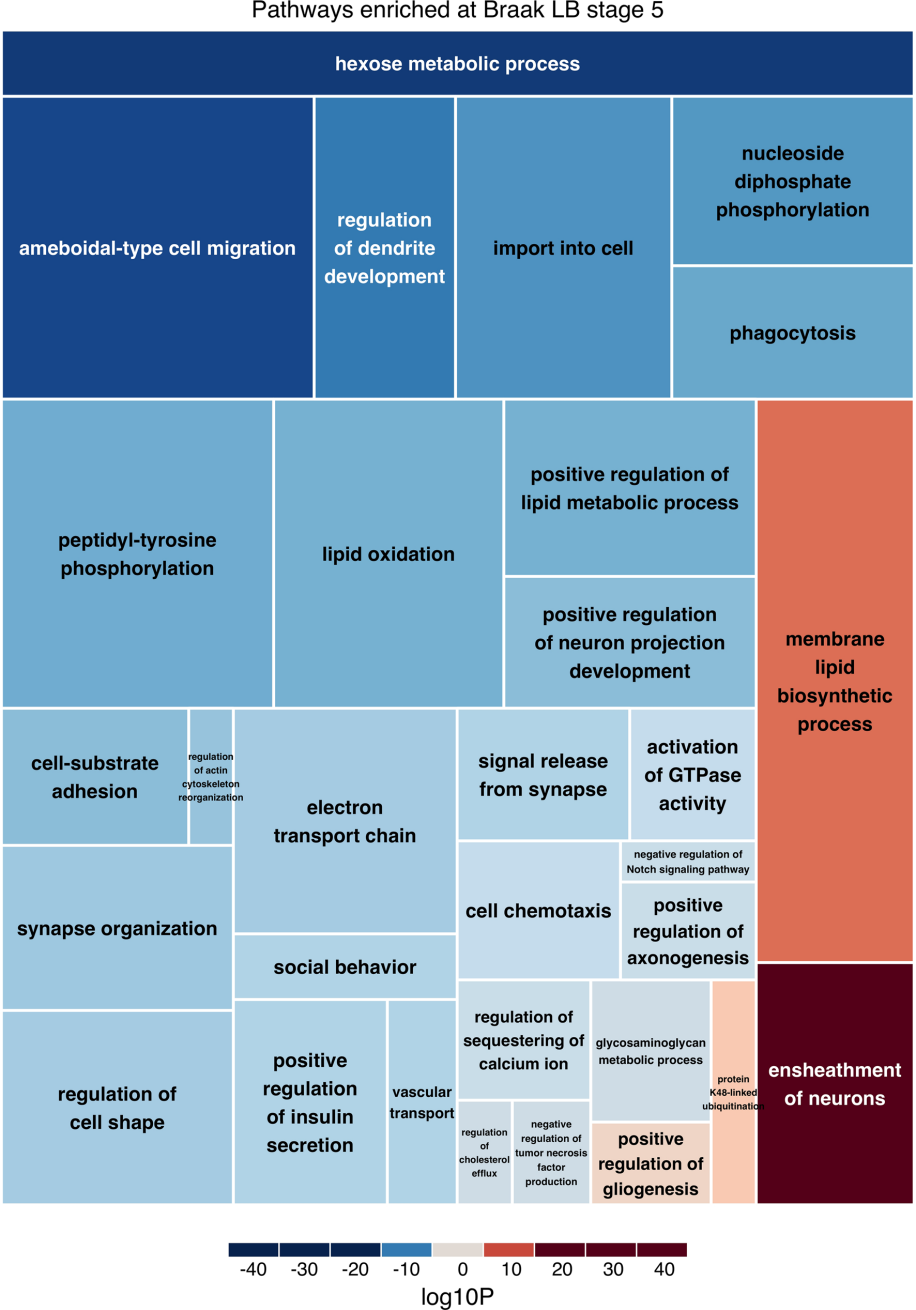
Pathways enriched at Braak LB stage 5. The treemap shows the significantly enriched pathways at Braak LB stage 5. Darker shades of blue/red represent lower enrichment *p*-values for down-/up-regulated pathways. The size of the rectangles is proportional to the number of genes included in each pathway

We performed functional enrichment analysis of the differential gene expression results obtained when comparing the individual neuropathological groups (1–4, 5 and 6) to the controls. We included all the 21,615 genes and assigned a score to each of them based on their *p*-value and log fold change for each comparison. With this method we found 99 Gene Ontology pathways significantly enriched (FDR < 0.05) from Braak LB stage 0 to 1–4 (Supplementary Table 6), 75 pathways significantly enriched when from Braak LB stage 0 to 5 (Supplementary Table 7) and 163 pathways significantly enriched when from Braak LB stage 0 to 6 (Supplementary Table 8). To further explore these results, we generated volcano plots highlighting the genes involved in the three most significant pathways enriched at each Braak LB stage (Supplementary Fig. 7). The volcano plots show that the direction of regulation of all pathways is driven by the majority of the genes involved in each pathway. This excludes the possibility that the pathway findings are driven by changes in the expression of single genes. When summarizing the Gene Ontology pathways, we found 29 pathways enriched at Braak LB stage 1–4, (Supplementary Fig. 8), 31 pathways enriched at Braak LB stage 5 (Fig. 5) and 39 pathways enriched at Braak LB stage 6 (Supplementary Fig. 9). Moreover, we found that 12 pathways (all down-regulated) were enriched at all Braak LB stages and they comprised pathways mainly involved in ATP metabolic processes (Table 1).

**Table 1:** Pathways enriched at all Braak LB stages

Name	ID	Corrected *p*-value 0 vs 14	Corrected *p*-value 0 vs 5	Corrected *p*-value 0 vs 6	Direction
ATP metabolic process	GO:0046034	6.35E-05	0.008566	0.002539	Down
Signal release from synapse	GO:0099643	0.0001715	0.02021101	0.02581261	Down
Glycolytic process	GO:0006096	0.0005491	0.0004233	1.14E-06	Down
Ribonucleoside triphosphate metabolic process	GO:0009205	0.0006046	0.03603505	0.02070463	Down
Purine ribonucleoside triphosphate metabolic process	GO:0009205	0.001119	0.01947014	0.009645	Down
Pyruvate metabolic process	GO:0006090	0.001384	0.002427	5.06E-07	Down
ATP generation from ADP	GO:0006757	0.001465	0.001017	5.10E-06	Down
Purine nucleoside diphosphate metabolic process	GO:0009135	0.01415693	0.008253	1.18E-05	Down
Purine ribonucleoside diphosphate metabolic process	GO:0009179	0.01415693	0.008253	1.18E-05	Down
Ribonucleoside diphosphate metabolic process	GO:0009185	0.02394241	0.02425647	5.85E-05	Down
ADP metabolic process	GO:0046031	0.02407688	0.002126	1.94E-05	Down
Carbohydrate catabolic process	GO:0016052	0.04027937	0.0006365	1.78E-05	Down

Pathways enriched at all Braak LB stages. The FDR corrected *p*-value for each group is shown in separate columns

### Risk genes

**Fig. 6:**
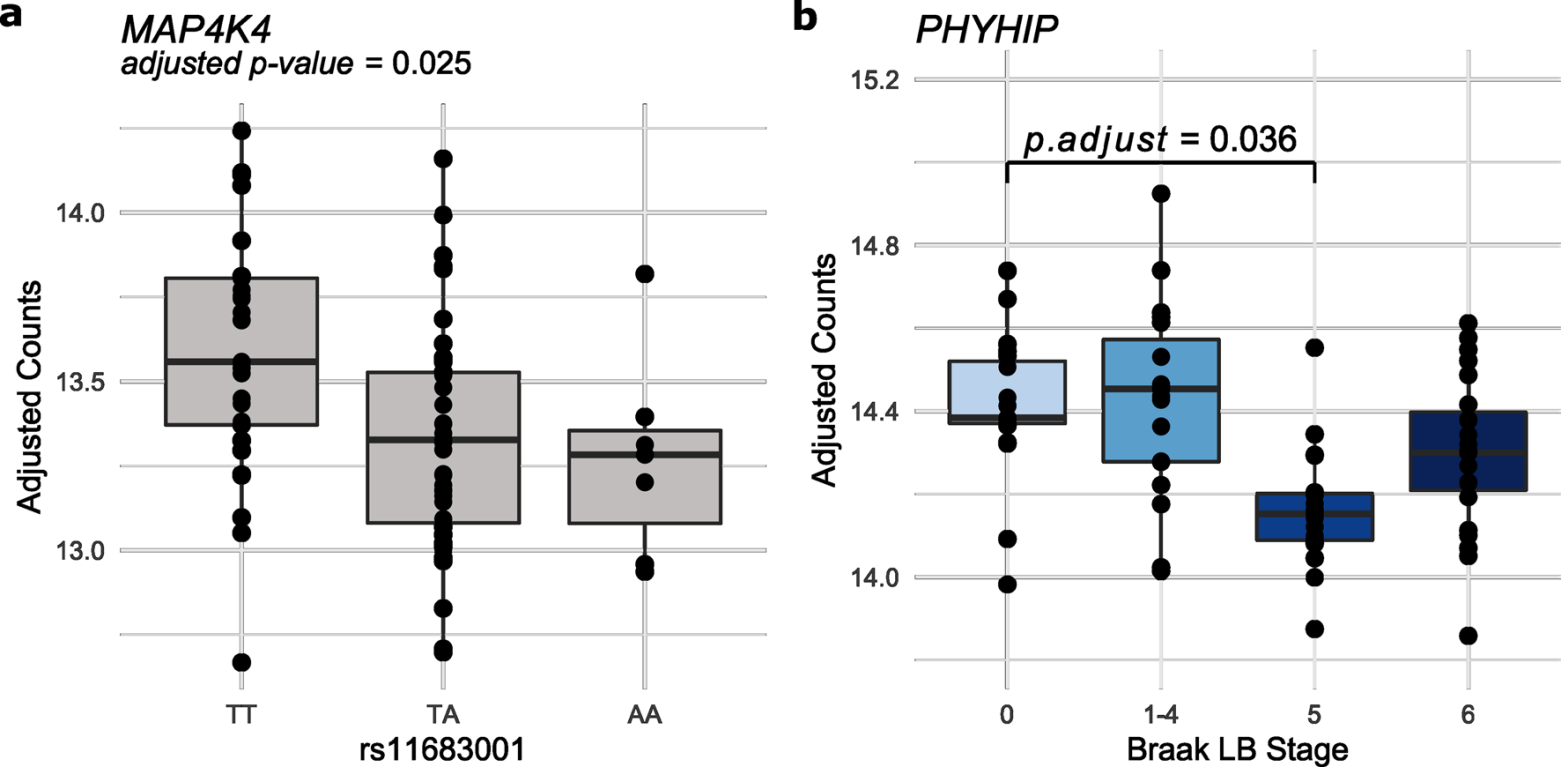
Risk genes. **a** *MAP4K4* expression by rs11683001 genotype. Samples were divided by their rs11683001 genotype and the adjusted counts for its neatest gene *MAP4K4* are plotted. **b** *PHYHIP* expression at each Braak LB stage

To investigate the functional and molecular mechanisms giving rise to the association between some SNPs and PD, we extracted the nearest gene and the QTL nominated gene to each of the 90 PD risk SNPs [60]. 87 of the 98 selected genes were expressed in our samples. Five were among the genes differentially expressed between Braak LB stage 0 and Braak LB stage 5 (Benjamini–Hochberg FDR < 0.05) (Table 2). This is not a statistically significant enrichment of PD genes among the differentially expressed genes at Braak LB stage 5 (Χ^2^ (1, *n* = 21,615) = 0.084, *p*-value = 0.773). To further investigate these genes, we performed eQTL analysis to evaluate whether the expression of these 5 genes was associated with the genotype of the nearby PD risk SNP. We found an association between rs11683001 and its nearest gene *MAP4K4* (adjusted *p*-value = 0.025) (Fig. 6a). Of note, none of the 87 risk genes expressed in our samples was among the genes differentially expressed between Braak LB stage 0 and Braak LB stage 6.

**Table 2:** Risk genes

Gene	Ensembl ID	SNP	CHR	BP	Risk allele	eQTL *p*-val	eQTL *p*-adj	log2 Fold Change	Wald *p*-val	Wald *p*-adj
PMVK	ENSG00000163344	rs114138760	1	154,898,185	c	0.738	0.883	− 0.180	0.0005	0.025
**MAP4K4**	**ENSG00000071054**	**rs11683001**	**2**	**102,396,963**	**a**	**0.005**	**0.025**	− **0.292**	**0.0014**	**0.038**
SCARB2	ENSG00000138760	rs6825004	4	77,110,365	c	0.816	0.883	0.213	0.0015	0.039
C5orf24	ENSG00000181904	rs11950533	5	134,199,105	a	0.883	0.883	0.149	0.0014	0.038
CHD9	ENSG00000177200	rs10221156	16	52,969,426	a	0.306	0.766	0.135	0.0009	0.032

Table listing five genes differentially expressed at Braak LB stage 5 and located near to a PD risk single nucleotide polymorphism (SNP). Expression quantitative trait locus *p*-value (eQTL *p*-val) and adjusted eQTL *p*-value (eQTL *p*-adj) are reported for each gene and corresponding SNP. The logarithmic fold change (log2 Fold Change) of each gene between Braak LB stage 0 and 5 is also included in addition to *p*-values (Wald *p*-val) and adjusted p-values (Wald *p*-adj) obtained in the differential expression analysis using the Wald test

We recently reported novel differentially methylated replicating loci associated with Braak LB stage near *TMCC2*, *SFMBT2*, *AKAP6* and *PHYHIP* [68]. We found that all four genes are expressed in our samples (Supplementary Fig. 10). One of these genes, *PHYHIP*, located near cg04011470 was also among the genes differentially expressed at Braak LB stage 5 (Fig. 6b).

## Discussion

We investigated transcriptomic changes in PD brains by comparing gene expression across different neuropathological disease stages to identify genes involved in the molecular processes associated with the development of LB pathology. We found major disease-stage specific transcriptomic alterations between the four groups that were examined. Studying frontal cortex, we found most pronounced changes in gene expression at Braak LB stage 5. In this disease stage, microscopic changes, i.e., the number of LBs in the superior frontal gyrus, are still moderate, while in stage 6 the LB pathology is more pronounced in the superior frontal gyrus, and also affecting the primary and secondary sensory and motor cortex. As the samples at Braak LB stage 5 included iLBD, PD and PDD individuals, this finding emphasizes the importance of taking pathological disease stage into account when designing and analyzing transcriptomic studies of neurodegenerative and other brain disorders. Further, the identified gene expression changes may provide new insights into the pathogenesis of the development of PD dementia, as many patients at this stage experience cognitive decline.

RNA quality and cell composition have been shown to be major confounders in transcriptomic studies of bulk tissue, and differences in cell-type proportions between controls and patients have been previously reported [1, 35, 63]. Therefore, prior to performing gene expression analysis, we used the deep-learning based deconvolution algorithm called Scaden [58] to predict cell-type proportions for our bulk-tissue RNA-seq samples. Our results support previous findings reporting an association between the RNA quality and cell-type proportions [63]. This finding is in accordance with the notion that, since some cell-types are more susceptible to degradation than others, the RNA quality can correlate with cell composition. Published studies also found differences in cell composition between the studied groups [35, 63]. After accounting for the quality of the RNA samples, we did not find any differences in cell-type proportions between the neuropathological groups, and therefore we did not take cell-type proportions into account in our gene expression analysis. This finding is not in accordance with a previous study employing the same method to calculate cell-type proportions [35]. However, this study had a much smaller sample size (7 controls, 7 PD, 7 PDD and 7 DLB) and used a brain region (anterior cingulate cortex) that is involved at an earlier stage of the disease and could therefore be more susceptible to cell composition changes compared to the superior frontal cortex used in our study [13]. No major neurodegeneration is generally observed for PD in the frontal cortex, as opposed to the *substantia nigra* used in several earlier transcriptomic, which undergoes major neurodegeneration in PD patients [65]. Additionally, we used a standardized and precise dissection method specifically aiming at avoiding differences in cell-type composition.

We acknowledge that the cell-type proportions we calculated in our study are only estimates and methods such as single-cell and single-nucleus RNA-seq could be used to overcome this limitation. However, it is challenging to perform brain scRNA-seq due to the intricate network of axons, dendrites and glial processes that are damaged and lost during tissue dissection and cell dissociation. Currently, RNA-seq of *postmortem* brain tissue at single cell resolution is limited to single-nucleus RNA-seq, were information about of 50–80% of the transcriptome is lost, including all transcripts that are expressed at a relatively low level [38]. Using information obtained from scRNA-seq to estimate bulk-tissue cell-type proportions, as we did, allows to account for cell composition without sacrificing part of the transcriptome. We therefore believe that RNA sequencing of bulk tissue from brain may provide important results that cannot be fully replaced by single-nucleus RNA-seq.

While all previous RNA-seq brain transcriptomic studies in PD [12, 26, 35, 45] focused on differences between clinically defined disease groups and non-neuropathological controls, we included 84 frontal cortex samples and we studied the transcriptome changes across their Braak LB stages. We categorized our samples according to their neuropathological stage instead of their clinical diagnosis, aiming at having a more objective measurement of progression. The study design was based on the concept of pathological stages of preclinical and clinical PD developed by Braak [13]. While this concept has been found valid for most cases of PD, it may not be universally applicable to all diseases with LB pathology [7]. Due to the differences in study design and in the methods used for data analyses between previous and our study the results are not easily comparable, however we note that many of the differentially expressed genes we identified were found differentially expressed also in a scRNA-seq study of PD, PDD and DLB [35]. We acknowledge that an even bigger sample size, possibly including several brain regions from the same donor, could have improved our study design. However, the use of standardized brain autopsies and neuropathological examinations with a very short PMD allowed us to obtain high quality samples and to exclude possible confounding due to differences in Alzheimer's pathology between groups.

In our gene expression analysis across all neuropathological groups, we found a total of 266 differentially expressed genes, of which 136 were also among the differentially expressed genes found in a previous scRNA-seq study of controls, PD, PDD and DLB patients [35]. We divided the differentially expressed genes across all neuropathological groups in 8 clusters based on their patterns of expression across the neuropathological groups. Two of these clusters showed a linear increase or decrease in expression. The top hit gene of the up-regulated cluster was *SNX7*. This gene has been shown to be differentially expressed in the excitatory neurons between PD and DLB patients [35]. *SNX7* encodes the sorting nexin 7 protein known to form a heterodimer with SNX4. Together these proteins are responsible of recruiting the phospholipid scramblase ATG9A to assemble a productive autophagosome and are therefore positive regulators of autophagy [5]. Both impairment and up-regulation of autophagy have been implicated in PD pathogenesis [40]. Our finding is in line with previous studies which suggested that, in PD models, upregulation of autophagy may act as a cellular compensatory mechanism to clear accumulated α-syn [47, 64]. Moreover, STRING [79] shows that *SNX7* is an interactor of *VPS29* and *VPS35.* The proteins encoded by these genes are part of the retromer complex and mutations in *VPS35* have been shown to cause late-onset, autosomal dominant familial PD [85]. Therefore, the increased *SNX7* expression observed throughout disease stages supports previous findings suggesting a role of the intracellular endosomal trafficking in PD and other neurodegenerative diseases [90].

The top hit gene of the down-regulated genes was *NUCB1*. In a previous study, *NUCB1* has been shown to be downregulated in the excitatory neurons and oligodendrocytes of patients [35]. This gene encodes the calcium-binding protein nucleobindin-1, which is a novel chaperone-like amyloid binding protein that inhibits aggregation of different amyloid proteins including α-syn [11]. Therefore, the decrease in expression of *NUCB1* could be one of the drivers of α-syn accumulation in LBs. Moreover, the protein encoded by *NUCB1* is a Golgi-resident protein with a putative DNA- and calcium-binding activity [82]. The other 6 clusters exhibited a pattern in which Braak LB stage 5 showed major changes in expression.

The top hit gene of cluster 2 showing a decrease in expression from the control group to the Braak LB stage 5 groups, followed by an increase at Braak LB stage 6, was *PDXK*. This gene was also among the differentially expressed genes identified in excitatory neurons and microglia by Feleke et al. [35]. This gene encodes the pyridoxal kinase protein involved in the conversion of vitamin B6 to pyridoxal-5-phosphate, an important cofactor in intermediary metabolism [57]. Low levels of vitamin B6 have been linked with an increased PD incidence in several studies [22, 59, 88]. Moreover, mutations in *PDXK* cause autosomal recessive axonal peripheral polyneuropathy [19], *PDXK* was among the genes differentially expressed in the substantia nigra of PD patients and a DNA variant (rs2010795) in this gene has been associated with an increased risk of PD [32]. Even though this association was ruled out in another study [39], our results suggest that low expression levels of *PDXK* observed at Braak LB stage 5 are linked to PD and could act through the same biological mechanism of vitamin B6.

The three most significant genes included in cluster 3, showing an increase in expression from the control group to the Braak LB stage 5 groups, followed by a decrease at Braak LB stage 6, are novel transcripts. This result, together with the fact that among the differentially expressed genes we found many genes encoding novel transcripts or lncRNAs, highlights the importance of including these transcripts in gene expression analysis. Many lncRNAs have been shown to be brain specific and their expression has been associated with neurodegenerative disorders, including PD [70]. However, the function of the majority of these lncRNAs has not been characterized yet. Our study identified lncRNAs that could be good candidates for further functional analysis that could help understanding the complex role that these transcripts have in PD pathogenesis.

Interestingly, when further investigating gene expression by comparing each neuropathological group to controls, we did not find any statistically differentially expressed gene at early disease stages, suggesting that no considerable transcriptional changes of single transcripts can be detected in the superior frontal cortex tissue before the appearance of LBs in this brain region. This was unexpected, as there is already loss of dopamine and frontal-striatal circuitry changes in early-stage PD. Moreover, we found 979 and 38 genes differentially expressed at Braak LB stage 5 and 6, respectively, with 28 genes being differentially expressed at both stages. 506 of the genes differentially expressed at Braak LB stage 5 were also among the genes identified by Feleke et al*.* in a scRNA-seq study [35]. Of note, *SNX7* was the top hit gene both at Braak LB stage 5 and 6. *PHYHIP* was among the genes differentially expressed at Braak LB stage 5. *PHYHIP* is highly expressed in brain [51] and differentially expressed in several cell types of PD, PDD and DLB patients [35]. This gene encodes the phytanoyl-Coa hydroxylase-interacting protein involved in protein localization [9]. Interestingly, *PHYHIP* is located in one (cg04011470) of the four differentially methylated loci associated with LB pathology that we recently identified [68] in a study that comprised the samples used here. In line with the increased methylation in the neuronal enhancer region located in *PHYHIP* exon 4, we observed a decrease in the expression of this gene in frontal cortex at Braak LB stage 5.

Among the genes differentially expressed at Braak LB stage 5 we also found 5 genes located within PD risk loci. When performing eQTL analysis for these genes we found an association between the rs11683001 and a decrease in the expression of its nearest gene *MAP4K4*. *MAP4K4* has been shown to be differentially expressed in several cell-types of PD, PDD and DLB patients [35]. This gene encodes a serine/threonine kinase that may play a role in the response to environmental stress and cytokines such as the tumor necrosis factor alpha [43] and it's activation has been shown to mediated motor neurons degeneration in amyotrophic lateral sclerosis [86]. However, further research is needed to understand the role that *MAP4K4* plays in PD.

Additionally, we found that pathways related to mitochondrial dysfunction and signal release from synapsis were enriched across all Braak LB stages. These pathway analyses are including transcripts also with small changes in gene expression, not considered significant in the differential expression analysis, and thus pathway changes may be found in tissue without significant alterations of single transcripts. The consistent downregulation of pathways involved in ATP metabolic process at all disease stages is noticeable and indicates that mitochondrial dysfunction may be an early event in the disease process. Pathways involved in ATP metabolic process are among the most consistent transcriptomic signatures in previous studies of PD [12, 29, 30], and mitochondrial dysfunction has been associated with neuronal loss and synaptic damage in early events of PD pathogenesis [72]. Moreover, inhibition of respiration through dysfunctions in complex I of the respiratory chain results in increased oxidative stress [62] and in reduced levels of nicotinamide adenine dinucleotide (NAD) [77, 81]. NAD replenishment therapy in newly diagnosed PD patients has shown potential neuroprotective effect in a randomized phase I trial [15].

Moreover, we identified mainly brain-specific enriched pathways at Braak LB stage 5. Several of these pathways are involved in axonal maintenance, suggesting that at this stage of the disease axonal dysfunction is an important feature. We found no differences in cell composition between our samples showing supporting previous studies showing that there is little neuronal cell loss in the cortex of patients with PD [37]. Therefore, the axonal dysfunction we observed may be a substrate for the increasing cognitive decline that patients develop at this disease stage. We also found that immune response pathways were significantly up-regulated at early disease stages and down-regulated at the most advanced stages of disease. It is known that neuroinflammation is associated with age-related neurodegenerative diseases such as PD [78]. However, neuroinflammation can have both beneficial and detrimental effects. Neuroinflammation can initially protect the brain by removing or inhibiting pathogens [87] or by promoting tissue repair. However, if sustained, it can inhibit regeneration [44]. Our findings suggest that inflammation is involved in the early stages of the disease, prior to the formation of LBs, rather than in later stages of the disease, as a result of cell disruption and the need to remove damaged cells. From our results it is still unclear whether this upregulation in the immune response is beneficial or destructive, nevertheless our results highlight the immune response as one of the possible targets for future disease modifying therapies. A limitation of pathway enrichment analysis is that these analyses are built on non-tissue specific available pathway databases and published data, biasing the results towards the chosen database. Therefore, further studies are needed to elucidate the involvement of the identified pathways in PD.

In summary, our transcriptomic analysis across Braak LB stages revealed that major changes in gene expression can be observed only when the LBs first appear in the studied diseased tissue whereas, before and after LBs' appearance, only minor changes can be detected. Furthermore, we confirmed the enrichment of pathways involved in ATP metabolic process and synaptic impairment throughout all stages of the disease. In contrast, pathways related to the immune response are up-regulated prior to the formation of LBs and then downregulated in later stages of the disease, highlighting the immune response as one of the possible targets for future disease modifying therapies. We also identified single genes (*SNX7, NUCB1, PDXK, PHYHIP* and *MAP4K4*) potentially involved in PD pathogenesis and pointed to a potential role of lncRNAs in disease pathogenesis.

## Supplementary Information

Below is the link to the electronic supplementary material.

Supplementary file1 (DOCX 53060 KB)

Supplementary file2 (XLSX 240 KB)

## Data Availability

RNA-sequencing data can be accessed through the Gene Expression Omnibus (accession ID: GSE216281). Code used to analyze RNA-sequencing data and to generate figures for the manuscript is available at: https://github.com/chiaracapp/Transcriptomic-profiling-of-PD-brains.git.
