# Supplementary material for: Transcriptomic profiling of Parkinson's disease brains reveals disease stage specific gene expression changes
Source: Acta Neuropathol. 2023 Jun 22;146(2):227–44. doi: 10.1007/s00401-023-02597-7 (PMC10329075; doi:10.1007/s00401-023-02597-7)
Supplement: Supplementary file 1 — Supplementary file1 (DOCX 53060 KB) [file 401_2023_2597_MOESM1_ESM.docx]

Transcriptomic profiling of Parkinson´s disease brains reveals disease stage specific gene expression changes

Chiara Cappelletti^1,2,3^, Sandra Pilar Henriksen^3^, Hanneke Geut^4,5^, Annemieke J.M. Rozemuller^6^, Wilma D.J. van de Berg^4^, Lasse Pihlstrøm^3^ and Mathias Toft^3,7^

^1^Department of Mechanical, Electronics and Chemical Engineering, Faculty of Technology, Art and Design, OsloMet – Oslo Metropolitan University, Oslo, Norway

^2^Department of Research, Innovation and Education, Oslo University Hospital, Oslo, Norway

^3^Department of Neurology, Oslo University Hospital, Oslo, Norway

^4^Amsterdam UMC, Vrije Universiteit, Section Clinical Neuroanatomy and Biobanking, Department of Anatomy and Neurosciences, Amsterdam Neuroscience, Amsterdam, Netherlands

^5^Netherlands Brain Bank, Netherlands Institute of Neurosciences, Amsterdam, Netherlands

^6^Amsterdam UMC, Vrije Universiteit, Department of Pathology, Amsterdam Neuroscience, Amsterdam, Netherlands

^7^Institute of Clinical Medicine, University of Oslo, Oslo, Norway

Corresponding author:

Mathias Toft, [mathias.toft@medisin.uio.no](mailto:mathias.toft@medisin.uio.no), telephone +47 99514189, fax +47 23074891

Journal name: Acta Neuropathologica

**Supplementary Figures**


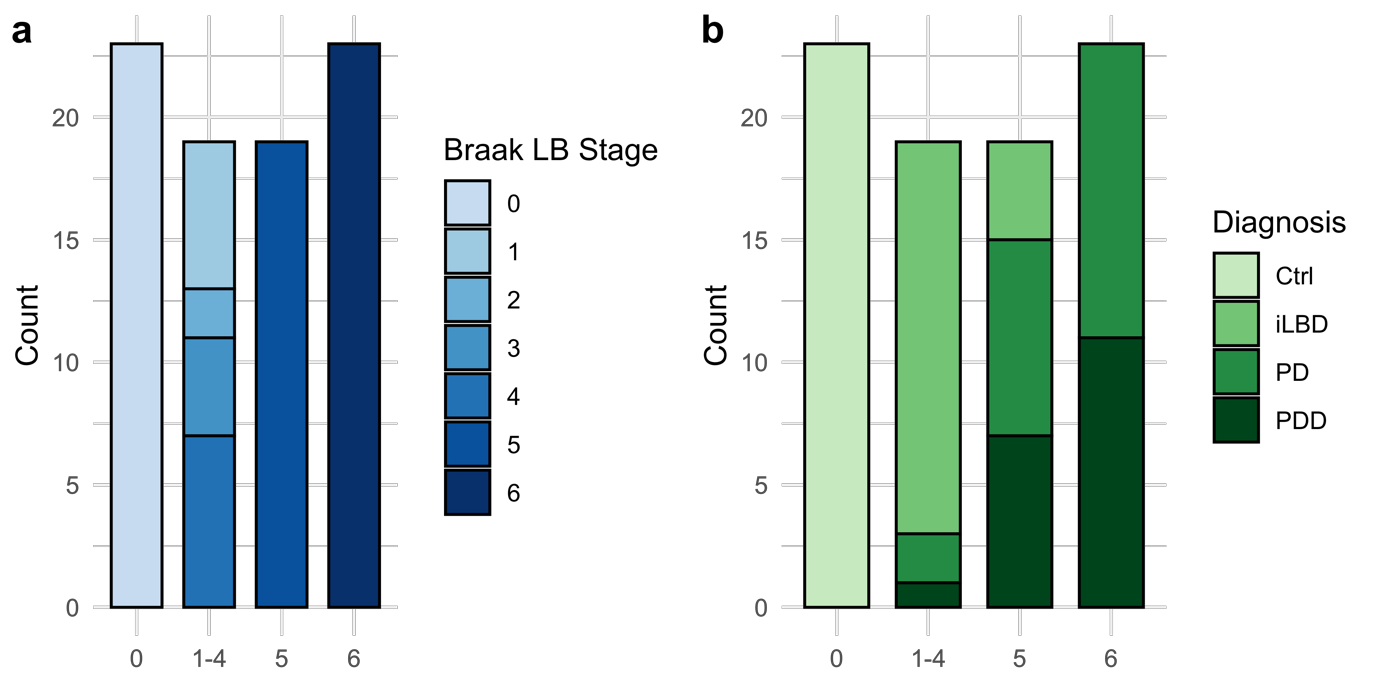


**Supplementary Fig. 1 Clinical diagnosis and Braak Lewy body stage of study individuals.** (a) This study included brain tissue from a total of 84 individuals: 23 non-neurological individuals and 61 individuals at different Braak LB stages. The latter were samples collected from donors with iLDB, PD or PDD and (b) were split into three neuropathological groups based on their Braak stage: one group consisting of individuals at Braak LB stages 1, 2, 3 and 4 (n = 19), one group consisting of individuals at Braak stage 5 (n = 19) and one group consisting of individuals at Braak stage 6 (n = 23). Ctrl, non-neurological individual; iLBD, incidental Lewy body disease; PD, Parkinson´s disease; PDD, Parkinson´s disease with dementia.


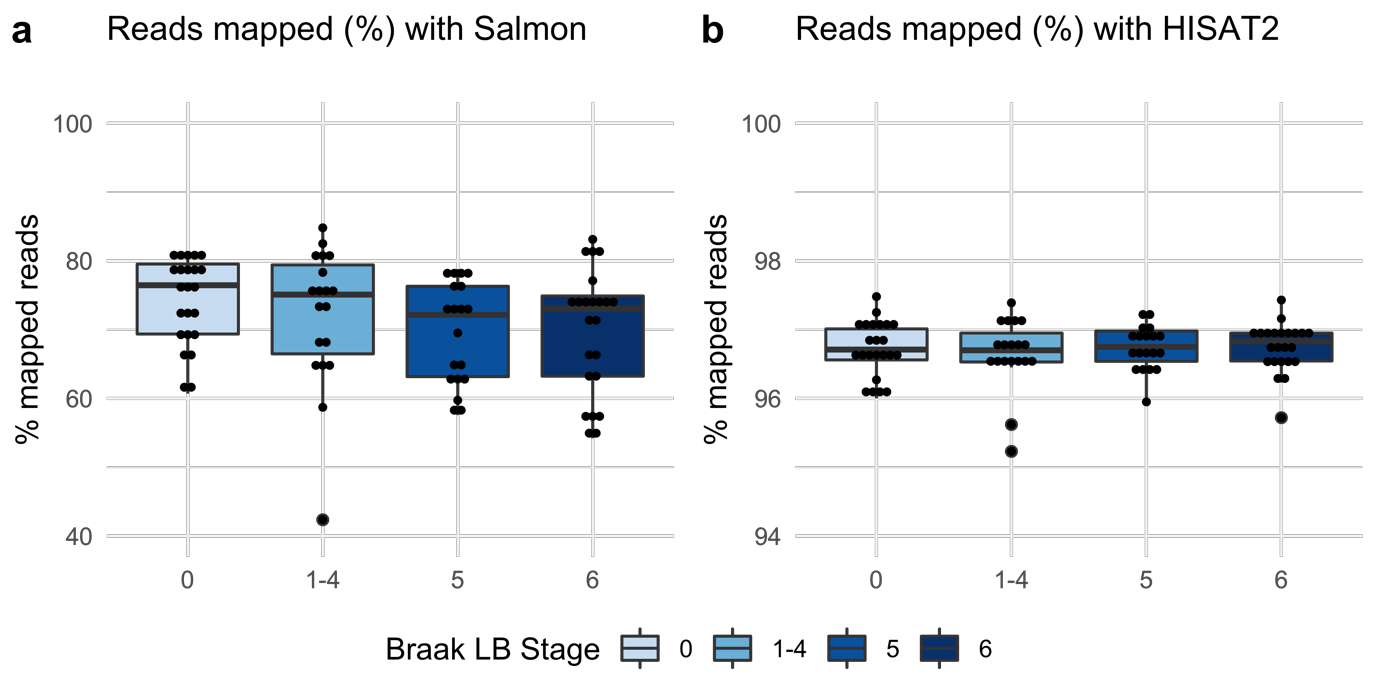


**Supplementary Fig. 2 Percentage of reads mapped with (a) Salmon and (b) HISAT2.**

**
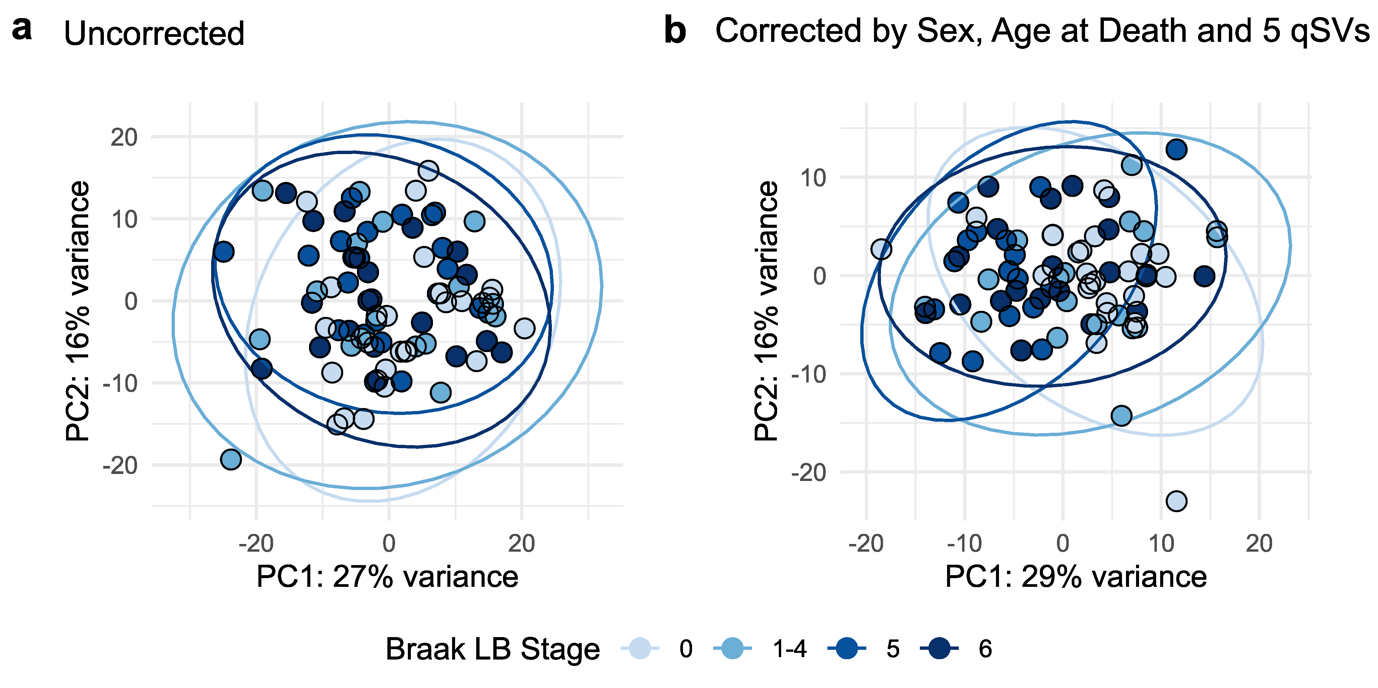
**

**Supplementary Fig. 3 Principal component analysis.** Samples plotted by the first two principal components derived from (a) uncorrected gene expression counts and (b) gene expression adjusted counts for sex, age at death and five qSVs. qSV, quality surrogate variable; PC, principal component.

**
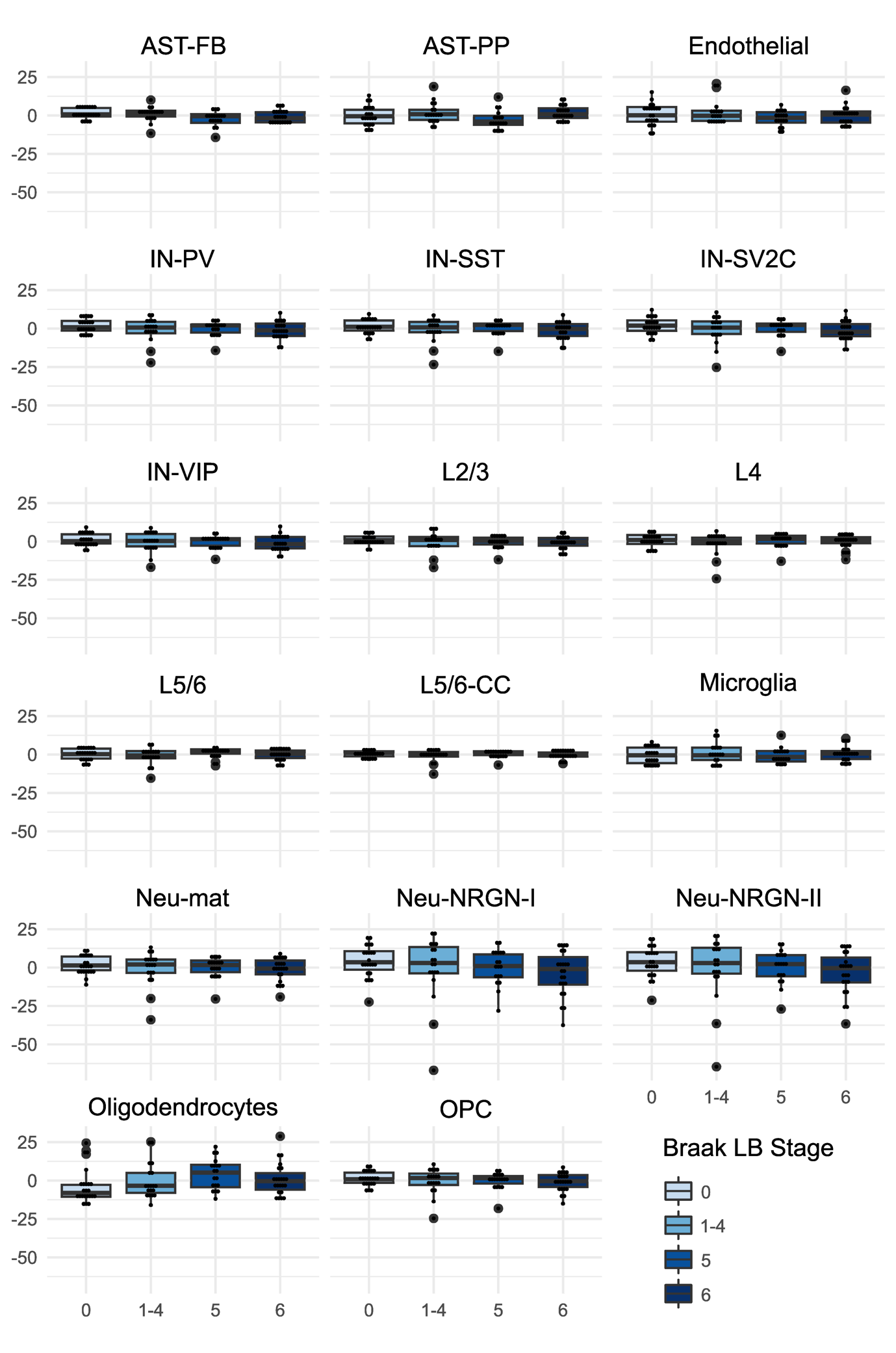
**

**Supplementary Fig. 4 Cell composition estimated with marker gene profiles.** AST-FB, fibrous astrocytes; AST-PP, Protoplasmic astrocytes; IN-PV, Parvalbumin interneurons; IN-SST, somatostatin interneurons; IN-SV2C, SV2C interneurons; IN-VIP, VIP interneurons; L2/3, layer 2/3 excitatory neurons; L4, layer 4 excitatory neurons, L5/6, layer 5/6 corticofugal projection neurons; L5/6-CC, layer 5/6 cortico-cortical projection neurons; Neu-mat, maturing neurons; Neu-NRGN-I, NRGN-expressing neurons I; Neu-NRGN-II, NRGN-expressing neurons II; OPC, oligodendrocyte precursor cells.

**
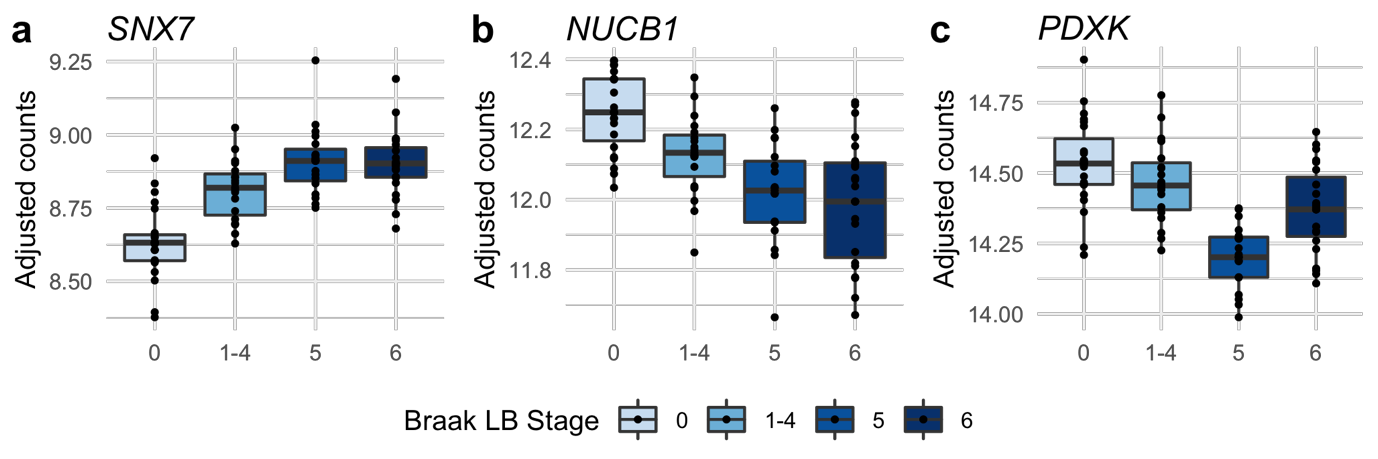
**

**Supplementary Fig. 5 Top hit genes in clusters 1, 4 and 2.** (a) Expression of top hit *SNX7* of cluster 1 at each Braak LB stage, (b) expression of top hit *NUCB1* of cluster 4 at each Braak LB stage and (c) expression of top hit *PDXK* of cluster 2 at each Braak LB stage.

**
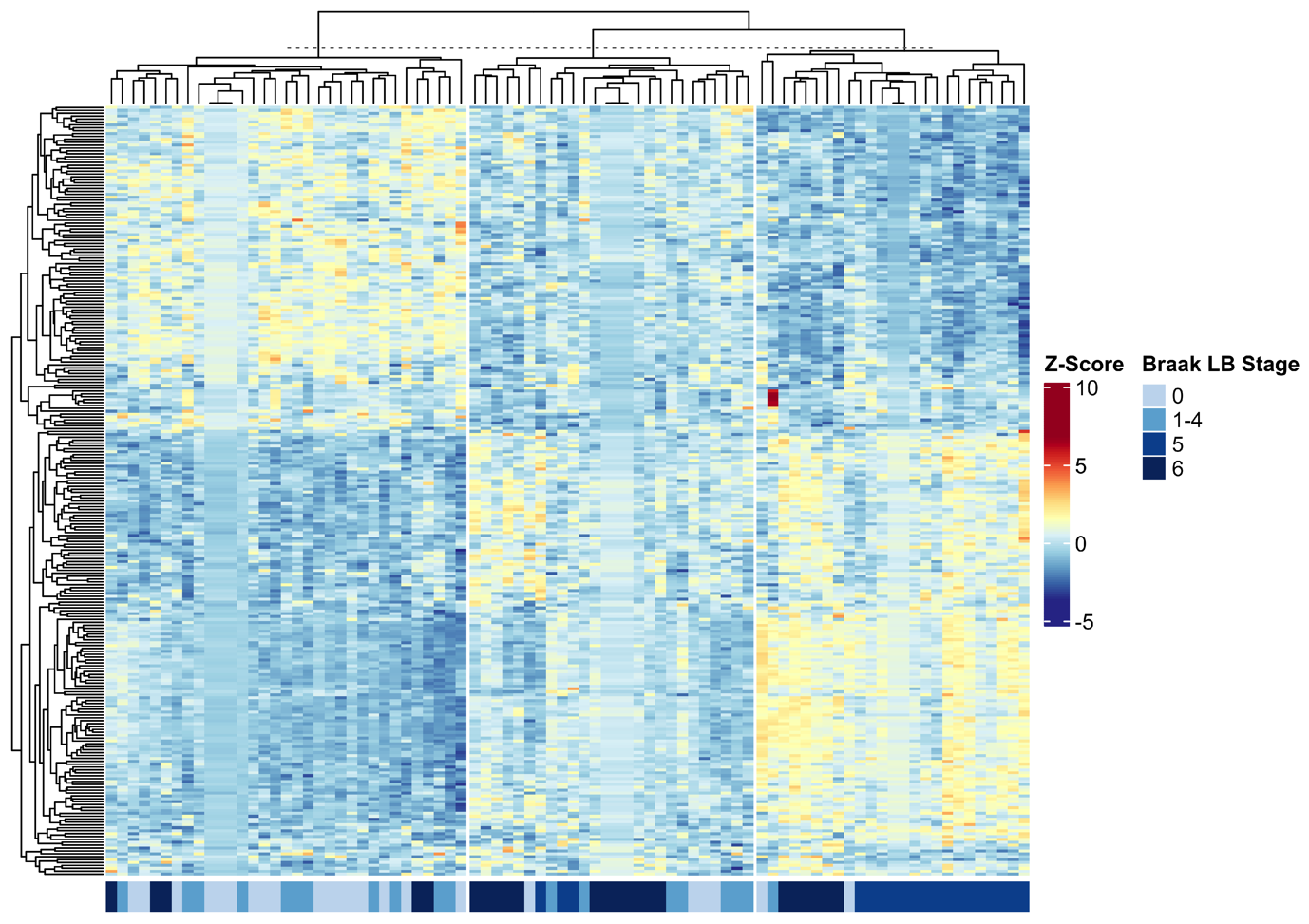
**

**Supplementary Fig. 6 Hierarchical clustering of samples.** The heatmap shows the clustering of the 84 samples based on their differential gene expression (Z-Score). The color scale shows genes that are up-regulated (red) or down-regulated (blue) relative to the mean expression of all samples. The bottom annotation shows to which neuropathological group each sample belongs. The main clade on the left is mainly composed of samples at Braak stage 5.


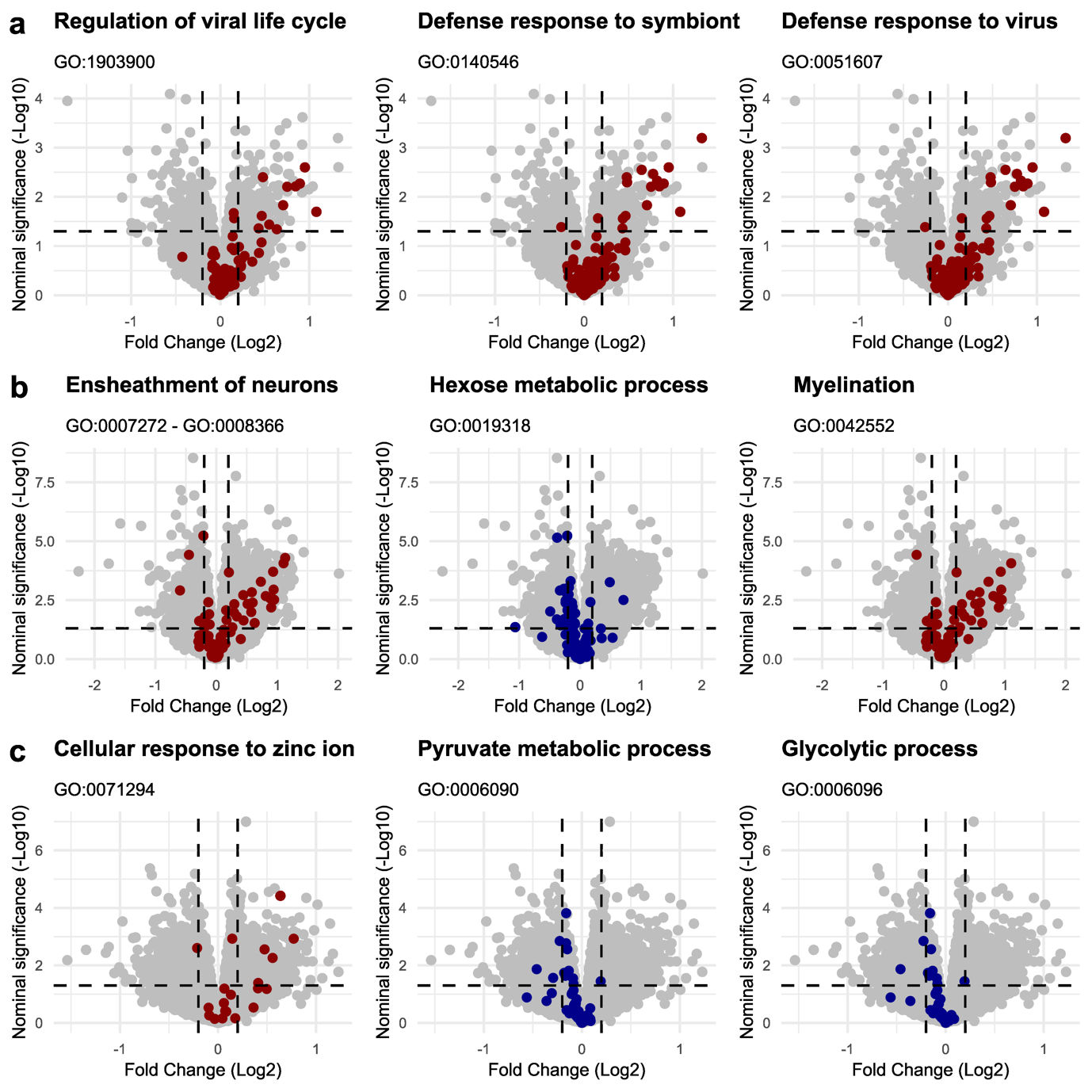


**Supplementary Fig. 7 Volcano plots showing the genes involved in the three most significant pathways enriched from Braak LB stage 0 to (a) Braak LB stage 1-4, (b) Braak LB stage 5 and (c) Braak LB stage 6.** The genes are highlighted in red in the up-regulated pathways and in blue in the down-regulated pathways.


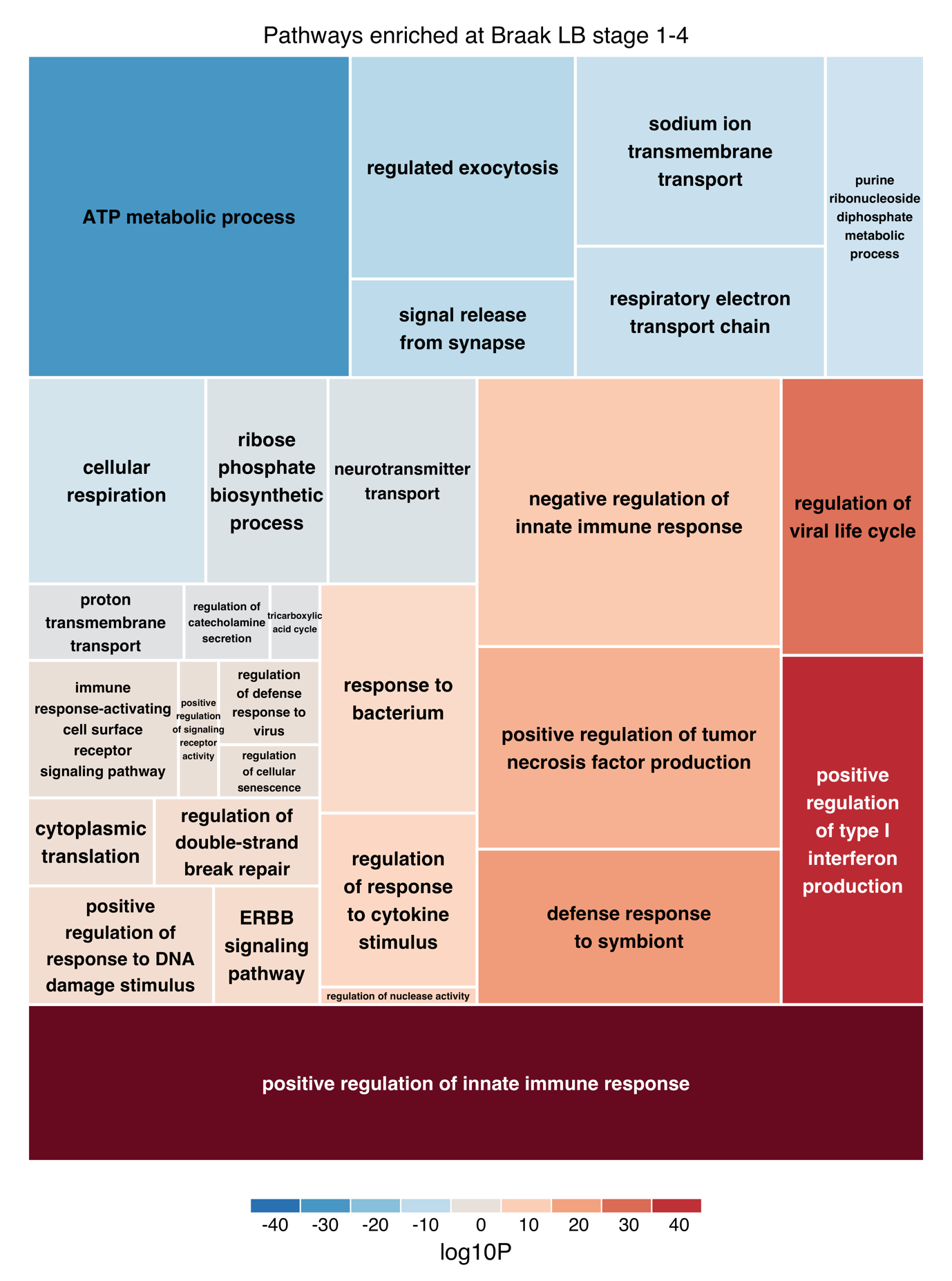


**Supplementary Fig. 8 Pathways enriched at Braak LB stage 1-4.** The treemap shows the significantly enriched pathways at Braak LB stage 1-4. Darker shades of blue/red represent lower enrichment p-values for down-/up-regulated pathways. The size of the rectangles is proportional to the number of genes included in each pathway.


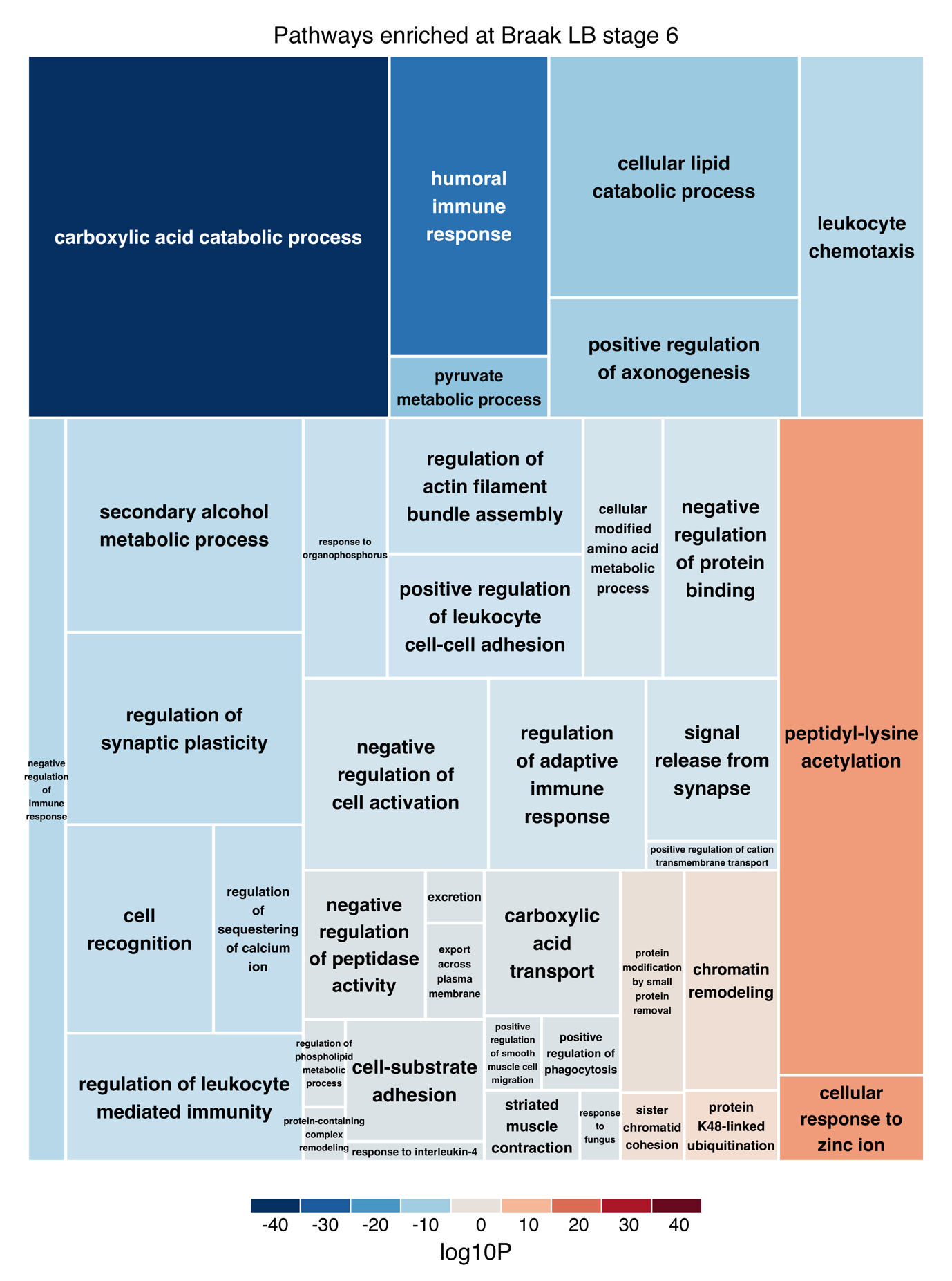


**Supplementary Fig. 9 Pathways enriched at Braak LB stage 6.** The treemap shows the significantly enriched pathways at Braak LB stage 6. Darker shades of blue/red represent lower enrichment p-values for down-/up-regulated pathways. The size of the rectangles is proportional to the number of genes included in each pathway.


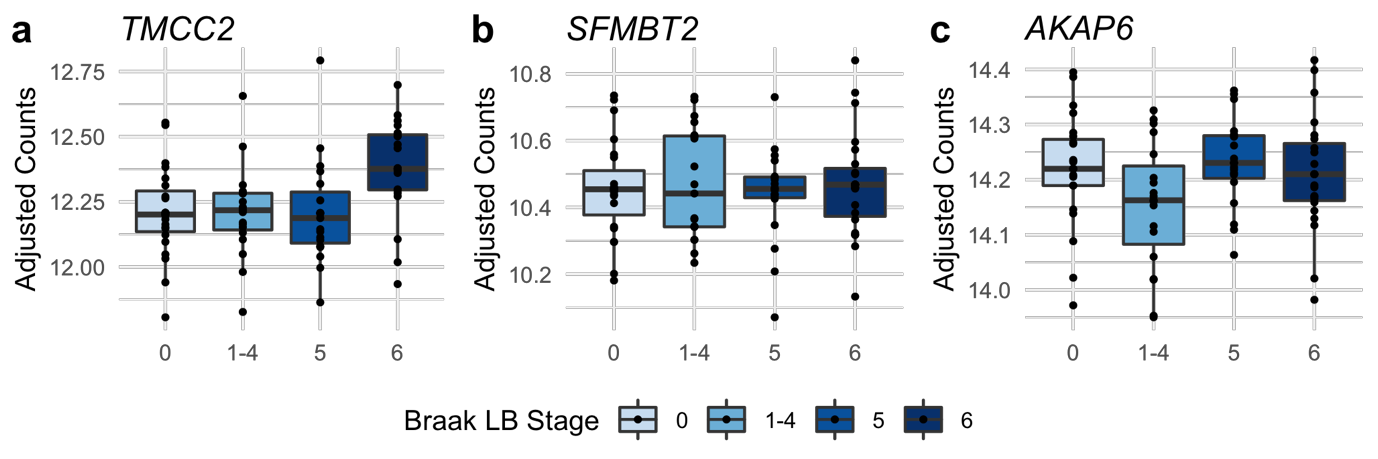


**Supplementary Fig. 10 Expression levels at each Braak LB stage of genes located near novel differentially methylated replicating loci associated with Braak LB stage.** (**a**) *TMCC2*, (**b**) *SFMBT2* and (**c**) *AKAP6*.

**Supplementary Text**

**Methods**

*DLB exclusion criterion*

DLB was diagnosed based on the presence of limbic-transitional or diffuse-neocortical Lewy pathology during autopsy, in combination with a clinical diagnosis of possible or probable DLB according to the consensus criteria of the DLB Consortium [3]. Distinction between DLB and PDD was made based on the 1-year rule, where dementia has to be present before or within one year of parkinsonism onset for the diagnosis of DLB [3]. In donors with dementia and a combination of LB and AD pathology, distinction between DLB and AD was made on the presence of neuropathological lesions, regardless of the clinical diagnosis, according to the consensus criteria of the DLB Consortium [3].

*Cell estimation using marker gene profiles*

It has been shown that each cell-type is characterized by the expression of specific genes that create a cell-type-specific transcriptional signature pattern. These patterns, called marker gene profiles (MGPs), can be obtained from scRNA-seq data and used to determine the cell-type composition of bulk RNA-seq samples [2]. We used a marker gene list obtained from scRNA-seq data of the human prefrontal cortex and anterior cingulate cortex [5] that comprises MGPs for 17 cell-types. Linear models adjusting for known experimental covariates (sex, age at death, PMD and RIN) were used to examine the differences in cell composition between the neuropathological groups. The p-values were adjusted for multiple testing using the Benjamini and Hochberg procedure.

*Hierarchical clustering*

Z-scores of the abundance of the significant genes (FDR < 0.05) were calculated using the zscore() function from the dtwclust R package [4]. Hierarchical clustering and plotting of the heatmap were performed using the Heatmap() function from the ComplexHeatmap R package [1].

**Results**

*Hierarchical clustering*

Hierarchical clustering of the differentially expressed genes confirmed the major changes in expression at Braak LB stage 5 as it demonstrated that our samples could be grouped in 3 clades of which one was mainly constituted by samples at Braak LB stage 5. The second clade was formed almost only by samples from groups 0 and 1-4, whereas the last clade was made by a large proportion of samples from group 6, but also by samples belonging to other neuropathological groups. This finding highlighted how gene expression in group 6 did not clearly distinguish it from the other groups as it did for group 5.

*Functional enrichment analysis*

We investigated the pathways unique in each neuropathological group and how their p-value changed from one Braak stage to the other. We found that 85 pathways lost significance (59 up-regulated and 26 down-regulated) and 61 pathways gained significance (9 up-regulated and 52 down-regulated) from Braak LB stage 1-4 to 5 (Supplementary Table 9). Among the pathways that lost significance from Braak LB stage 1-4 to Braak LB stage 5 there were up-regulated pathways mainly implicated in the immune response and down-regulated pathways involved in signal release from synapses, neurotransmitter transport and cellular respiration. Instead, among the pathways that gained significance from Braak LB stage 1-4 to Braak LB stage 5 there were up-regulated pathways associated with axon ensheathment and membrane lipid biosynthetic process and down-regulated pathways involved in the hexose metabolic process, the regulation of dendrite development and the transport across the blood-brain barrier. When looking at the unique pathways enriched at Braak LB stage 5 or 6, we found 43 pathways that lost significance (8 up-regulated and 35 down-regulated) and 131 that gained significance (20 up-regulated and 111 down-regulated) from Braak LB stage 5 to Braak LB stage 6 (Supplementary Table 10). Among those that lost significance there were up-regulated pathways implicated in the ensheathment of neurons and in the membrane lipid biosynthetic process, and down-regulated pathways associated with regulation of dendrite development. Lastly, among the pathways that gained significance from Braak LB stage 5 to Braak LB stage 6 we found up-regulated pathways involved in protein acetylation and in the regulation of RNA splicing, and down-regulated pathways associated with the adaptive and humoral immune response among others.

**Supplementary Tables**

**Supplementary Table 1 Individuals´ information.** Ctrl, non-neurological individual; iLBD, incidental Lewy body disease; PD, Parkinson´s disease; PDD, Parkinson´s disease with dementia; PMD, postmortem delay; RIN, RNA integrity number; NFT, neurofibrillary tangle; CERAD, consortium to establish a registry for Alzheimer´s disease; AD, Alzheimer´s disease; CAA, cerebral amyloid angiopathy.

**Supplementary Table 2 Cell-type proportions.** Cell-type proportions for each sample predicted using Scaden. VLMC, vascular and leptomeningeal cell; OPC; oligodendrocyte progenitor cell; NFO, newly formed oligodendrocyte.

**Supplementary Table 3 Differentially expressed genes across Braak LB stages.** Ensemble_id, Gene Ensembl ID; hgnc_symbol, gene HGNC symbol; baseMean, mean of normalized counts for all samples; stat, test statistic (test used = likelihood ratio test); pvalue, p-value; padj, FDR-adjusted p-value; Cluster, number of the cluster in which each gene has been group during pattern analysis.

**Supplementary Table 4 Differentially expressed genes between Braak LB stage 0 and Braak LB stage 5.** Ensemble_id, Gene Ensembl ID; hgnc_symbol, gene HGNC symbol; baseMean, mean of normalized counts for all samples; log2FoldChange, log2 fold change (using DESeq2 maximum likelihood estimation) the reference group is Braak stage 0; lfcSE, log2 fold change standard error; stat, test statistic (test used = Wald test); pvalue, p-value; padj, FDR-adjusted p-value.

**Supplementary Table 5 Differentially expressed genes between Braak LB stage 0 and Braak LB stage 6.** Ensemble_id, Gene Ensembl ID; hgnc_symbol, gene HGNC symbol; baseMean, mean of normalized counts for all samples; og2FoldChange, log2 fold change (using DESeq2 maximum likelihood estimation) the reference group is Braak stage 0; lfcSE, log2 fold change standard error; stat, test statistic (test used = Wald test); pvalue, p-value; padj, FDR-adjusted p-value.

**Supplementary Table 6 Pathways significantly enriched between Braak LB stage 0 and Braak LB stage 1-4.**

**Supplementary Table 7 Pathways significantly enriched between Braak LB stage 0 and Braak LB stage 5.**

**Supplementary Table 8 Pathways significantly enriched between Braak LB stage 0 and Braak LB stage 6.**

**Supplementary Table 9 Pathways that gain or lose significance from Braak LB stage 1-4 to Braak LB stage 5.**

**Supplementary Table 10 Pathways that gain or lose significance from Braak LB stage 5 to Braak LB stage 6.**

**References:**

1 Gu Z, Eils R, Schlesner M (2016) Complex heatmaps reveal patterns and correlations in multidimensional genomic data. Bioinformatics 32: 2847-2849 Doi 10.1093/bioinformatics/btw313

2 Mancarci BO, Toker L, Tripathy SJ, Li B, Rocco B, Sibille E, Pavlidis P (2017) Cross-Laboratory Analysis of Brain Cell Type Transcriptomes with Applications to Interpretation of Bulk Tissue Data. eNeuro 4: Doi 10.1523/ENEURO.0212-17.2017

3 McKeith IG, Galasko D, Kosaka K, Perry EK, Dickson DW, Hansen LA, Salmon DP, Lowe J, Mirra SS, Byrne EJet al (1996) Consensus guidelines for the clinical and pathologic diagnosis of dementia with Lewy bodies (DLB): report of the consortium on DLB international workshop. Neurology 47: 1113-1124 Doi 10.1212/wnl.47.5.1113

4 Sarda-Espinosa A (2019) dtwclust: Time series clustering along with optimizations for the dynamic time warping distance. R package version 5:

5 Velmeshev D, Schirmer L, Jung D, Haeussler M, Perez Y, Mayer S, Bhaduri A, Goyal N, Rowitch DH, Kriegstein AR (2019) Single-cell genomics identifies cell type-specific molecular changes in autism. Science 364: 685-689 Doi 10.1126/science.aav8130
